# Explainable Machine Learning Model for Predicting Persistent Sepsis-Associated Acute Kidney Injury: Development and Validation Study

**DOI:** 10.2196/62932

**Published:** 2025-04-28

**Authors:** Wei Jiang, Yaosheng Zhang, Jiayi Weng, Lin Song, Siqi Liu, Xianghui Li, Shiqi Xu, Keran Shi, Luanluan Li, Chuanqing Zhang, Jing Wang, Quan Yuan, Yongwei Zhang, Jun Shao, Jiangquan Yu, Ruiqiang Zheng

**Affiliations:** 1 Department of Critical Care Medicine Northern Jiangsu People's Hospital Affiliated to Yangzhou University Yangzhou China; 2 School of Clinical and Basic Medicine Shandong First Medical University & Shandong Academy of Medical Sciences Jinan China; 3 School of Economics and Management Beijing Jiao Tong University Beijing China

**Keywords:** sepsis, persistent acute kidney injury, machine learning, prediction model, Shapley Additive Explanations

## Abstract

**Background:**

Persistent sepsis-associated acute kidney injury (SA-AKI) shows poor clinical outcomes and remains a therapeutic challenge for clinicians. Early identification and prediction of persistent SA-AKI are crucial.

**Objective:**

The aim of this study was to develop and validate an interpretable machine learning (ML) model that predicts persistent SA-AKI and to compare its diagnostic performance with that of C-C motif chemokine ligand 14 (CCL14) in a prospective cohort.

**Methods:**

The study used 4 retrospective cohorts and 1 prospective cohort for model derivation and validation. The derivation cohort used the MIMIC-IV database, which was randomly split into 2 parts (80% for model construction and 20% for internal validation). External validation was conducted using subsets of the MIMIC-III dataset and e-ICU dataset, and retrospective cohorts from the intensive care unit (ICU) of Northern Jiangsu People’s Hospital. Prospective data from the same ICU were used for validation and comparison with urinary CCL14 biomarker measurements. Acute kidney injury (AKI) was defined based on serum creatinine and urine output, using the Kidney Disease: Improving Global Outcomes (KDIGO) criteria. Routine clinical data within the first 24 hours of ICU admission were collected, and 8 ML algorithms were used to construct the prediction model. Multiple evaluation metrics, including area under the receiver operating characteristic curve (AUC), were used to compare predictive performance. Feature importance was ranked using Shapley Additive Explanations (SHAP), and the final model was explained accordingly. In addition, the model was developed into a web-based application using the Streamlit framework to facilitate its clinical application.

**Results:**

A total of 46,097 patients with sepsis from multiple cohorts were enrolled for analysis. Among 17,928 patients with sepsis in the derivation cohort, 8081 patients (45.1%) showed progression to persistent SA-AKI. Among the 8 ML models, the gradient boosting machine (GBM) model demonstrated superior discriminative ability. Following feature importance ranking, a final interpretable GBM model comprising 12 features (AKI stage, ΔCreatinine, urine output, furosemide dose, BMI, Sequential Organ Failure Assessment score, kidney replacement therapy, mechanical ventilation, lactate, blood urea nitrogen, prothrombin time, and age) was established. The final model accurately predicted the occurrence of persistent SA-AKI in both internal (AUC=0.870) and external validation cohorts (MIMIC-III subset: AUC=0.891; e-ICU dataset: AUC=0.932; Northern Jiangsu People’s Hospital retrospective cohort: AUC=0.983). In the prospective cohort, the GBM model outperformed urinary CCL14 in predicting persistent SA-AKI (GBM AUC=0.852 vs CCL14 AUC=0.821). The model has been transformed into an online clinical tool to facilitate its application in clinical settings.

**Conclusions:**

The interpretable GBM model was shown to successfully and accurately predict the occurrence of persistent SA-AKI, demonstrating good predictive ability in both internal and external validation cohorts. Furthermore, the model was demonstrated to outperform the biomarker CCL14 in prospective cohort validation.

## Introduction

Acute kidney injury (AKI) is a common and severe complication in critically ill patients, with sepsis being the most frequent cause [1]. AKI occurring within 7 days after the onset of sepsis is defined as sepsis-associated AKI (SA-AKI) [2]. Studies have estimated that 68% of patients with sepsis present with AKI on admission, 40% present with severe AKI, and 27% require subsequent kidney replacement therapy (KRT) during their intensive care unit (ICU) stay [3]. The development of SA-AKI correlates significantly with higher mortality rates and an increased risk of chronic kidney disease (CKD) [4,5].

SA-AKI is a complex clinical syndrome, and depending on interactions between genotypes and exposures, SA-AKI can lead to various clinical phenotypes. This heterogeneity complicates efficacy assessments in clinical trials of sepsis interventions because different treatments may only be beneficial for specific disease subtypes [2]. Recently, the Acute Disease Quality Initiative (ADQI) 16 Workgroup suggested that AKI be classified as transient (complete reversal of AKI within 48 hours) or persistent (continuance of AKI beyond 48 hours) [6]. Compared to transient AKI, persistent AKI is associated with ongoing host response dysregulation and adverse outcomes in critically ill patients with sepsis [7].

Early identification of persistent AKI has significant implications for risk stratification and individualized treatment. First, the duration of AKI closely relates to patient prognosis and the risk of end-stage renal failure. Recent evidence shows that two-thirds of all cases recover kidney function within 3 to 7 days, while those with persistent conditions show significantly reduced survival rates after 1 year [8]. Additionally, continued presence also increases the risk of CKD [9]. Early recognition and proactive intervention could potentially influence progression toward CKD. Second, the duration of AKI is closely related to the need for KRT. Studies have shown that some patients may benefit from starting KRT earlier, while others might not require it at all, as they can quickly recover kidney function [10]. Therefore, predicting the short-term reversibility of AKI could help assess the potential need for KRT and determine the optimal timing for its initiation.

However, due to complex mechanisms potentially co-existing in SA-AKI pathophysiology, including circulatory dysfunction, inflammatory response, mitochondrial dysfunction, and metabolic reprogramming [1], the single-method diagnosis of persistent SA-AKI has limited effectiveness. Traditional urinary indicators and renal ultrasound markers reportedly play limited roles in distinguishing between transient and persistent AKI [11-14]. Furthermore, most biomarkers assessing functional or injury value perform poorly in predicting persistent AKI [15-18], while others still require further clinical validation [19,20], with none currently approved for clinical use. There remains a lack of effective methods for rapidly identifying potential cases of persistent SA-AKI.

In recent years, machine learning (ML) methods derived from electronic medical records (EMRs) have gained attention and recognition among clinicians. The widespread application of EMRs within hospitals has made patient data collection more accurate and convenient. Currently, some studies suggest that predictive models built using ML methods are promising assessment tools for AKI [21-26]. Although these models demonstrate high diagnostic performance, they do not differentiate between clinical subtypes, which limits their guidance on personalized treatment strategies. Additionally, ML models remain difficult to interpret directly, presenting what is known as the “black box” problem, which limits their application by clinicians. The study by Jiang et al [27] used an ML method to build a predictive model, but they did not target patients with sepsis, and the model was not externally validated.

In our study, we developed an interpretable ML model in 4 retrospective cohorts and 1 prospective cohort, aimed at early and accurate prediction of persistent sepsis-associated AKI. We elucidated the importance of characterization and interpreted the model using the Shapley Additive Explanations (SHAP) method while comparing it to an existing biomarker, C-C motif chemokine ligand 14 (CCL14), which has high diagnostic performance.

## Methods

### Data Source and Study Population

The derivation cohort and internal validation cohort originated from the MIMIC-IV database. The MIMIC-IV database is a publicly available multiparameter intensive care database provided by Massachusetts Institute of Technology (MIT) [28]. It includes critically ill patients admitted to the ICU at Beth Israel Deaconess Medical Center in Boston, Massachusetts, from 2008 to 2019.

The datasets used for external validation include the MIMIC-III dataset, the e-ICU dataset, and 2 cohorts of patients admitted to the ICU of Northern Jiangsu People’s Hospital. The external validation cohort 1 is a subset of the MIMIC-III dataset. The MIMIC-III database is a publicly available multiparameter intensive care database provided by MIT. It includes critically ill patients admitted to the ICU at Beth Israel Deaconess Medical Center in Boston, Massachusetts, from 2008 to 2012 [29]. Considering that the MIMIC-III dataset and the MIMIC-IV dataset have overlapping sections, we only included the population from this dataset between 2001 and 2008. The external validation cohort 2 consists of patients with sepsis from the e-ICU database. The e-ICU Collaborative Research Database is a multicenter database comprising deidentified health data associated with over 200,000 admissions to ICUs across the United States between 2014 and 2015. The database includes vital sign measurements, care plan documentation, severity of illness measures, diagnosis information, and treatment information. Data are collected through the Philips e-ICU program, a critical care telehealth program that delivers information to caregivers at the bedside [30]. The external validation cohort 3 is a retrospective cohort of patients admitted to the ICU at Northern Jiangsu People’s Hospital from October 2021 to January 2023. The external validation cohort 4 is a prospective cohort of patients admitted to the ICU at Northern Jiangsu People’s Hospital from October 2023 to March 2024, where urine samples were collected upon their admission and CCL14 levels were measured using enzyme-linked immunosorbent assay (ELISA) kits.

The development and validation cohorts adopted identical inclusion and exclusion criteria. We included adult patients who developed sepsis after their first admission to the ICU and excluded those with stage 4-5 CKD.

### Ethical Considerations

Some of the data in this study are based on a third-party anonymous public database, for which we have already obtained approval from the Institutional Review Board (approval number: 54780440). Therefore, this portion of data does not require ethical review. In order to access this database, we completed an online training course and examination for protecting human research participants provided by the US National Institutes of Health (ID number: 54780440). The data collected by the ICU at Northern Jiangsu People’s Hospital have been approved by its Ethics Committee (ID number: 2021ky230) and registered on the China Clinical Trial Registration Platform (registration number: ChiCTR2100050540).

### Primary Outcomes and Definitions

The study outcome was the occurrence of persistent SA-AKI. Persistent SA-AKI was defined as the development of SA-AKI upon admission to the ICU and its duration exceeding 48 hours. Patients who died or started KRT within 48 hours after developing SA-AKI were also considered to have persistent SA-AKI [6].

According to the definition of Sepsis-3, we identified patients with confirmed or suspected infection and a Sequential Organ Failure Assessment (SOFA) score increase of 2 or more [31]. We determined the clinician’s recognition of suspected infection through two simultaneous events in the EHRs: (1) prescription of antibiotics and (2) ordering specific fluid cultures. These 2 events need to occur within a specific time frame, and there are 2 options. In option 1, fluid culture is performed first, and antibiotic use must be initiated within 72 hours. In option 2, antibiotics are administered first, and fluid culture must be completed within 24 hours. We excluded all antibiotics given as a single dose in the operating room. We also excluded antibiotics that were not accompanied by fluid cultures. We assumed a SOFA score of 0 prior to ICU admission. If individual components of SOFA were missing, no contribution was made to the total score [32]. The daily total SOFA scores were calculated, and an increase of 2 points within 24 hours was considered abnormal [33]. Considering difficulties in interpreting the neurological SOFA score when sedation therapy is being concurrently administered, it was not included in the overall scoring category [34].

The diagnostic criteria for AKI follow the Kidney Disease: Improving Global Outcomes (KDIGO) standards. The criteria were as follows: an increase in serum creatinine (sCr) exceeding 26.5 μmol/L (0.3 mg/dL) within 48 hours; an increase in sCr by more than 50% from baseline, lasting for 7 days; and urine output less than 0.5 mL/(kg·h), lasting for more than 6 hours [35]. Baseline sCr was defined as the lowest sCr value during 7 days before ICU admission [36,37]. When preadmission sCr was not available, baseline creatinine was estimated using the MDRD (Modification of Diet in Renal Disease) equation by back calculation from an estimated glomerular filtration rate (GFR) of 75 mL/min/1.73 m^2^ [38]. Urine-based or creatinine-based criteria, or a combination of both criteria were used to determine if a patient meets the KDIGO AKI criteria.

After determining sepsis and AKI separately, we applied the definition of SA-AKI from the ADQI 28 Working Group. We compared the diagnosis day of sepsis with the diagnosis day of AKI. If AKI occurred within 1-7 days after the diagnosis of sepsis, patients were considered to have SA-AKI according to the ADQI criteria [2]. If AKI occurred before sepsis, patients did not meet the definition of SA-AKI.

### Data Collection and Processing

We used demographic characteristics, vital sign measurements, and laboratory data collected within the first day of ICU admission to identify features and construct a predictive model (Multimedia Appendix 1). All data were sourced from EMRs. Due to potential multicollinearity among features that could affect prediction accuracy, any feature showing high correlation (correlation coefficient >0.6) with another in Spearman correlation analysis was removed if it had less relevance to the outcome (Multimedia Appendix 2). Additionally, we excluded any feature missing more than 30% of its values in subsequent analyses to minimize bias due to missing data. To normalize the data, we used the StandardScaler z-score normalization function. To process categorical features, we used label encoding. Given the large number of categorical variables in our dataset, label encoding was chosen over one-hot encoding to mitigate the dimensionality expansion problem and thereby improve computational efficiency.

Finally, we developed our predictive model using 58 features, including age, gender, BMI, maximum heart rate, minimum mean arterial pressure, maximum respiratory rate, highest temperature, maximum blood glucose level, AKI stage, 24-hour creatinine change, medical history (hypertension, diabetes, coronary heart disease, CKD, chronic heart failure, chronic liver disease, chronic cardiac disease, and chronic obstructive pulmonary disease), source of infection (pulmonary infection, blood infection, urinary tract infection, abdominal infection, skin infection, and others), interventional treatment (use of mechanical ventilation, KRT, diuretics, statins, angiotensin-converting enzyme inhibitors/angiotensin receptor blockers, aminoglycoside antibiotics, glycopeptide antibiotics, nonsteroidal anti-inflammatory drugs, and acyclovir antiviral drugs), furosemide dosage, urine output, maximum pH value, maximum lactate value, 24-hour lactate clearance rate, minimum arterial oxygen tension value (PaO2), 24-hour oxygen tension change value, maximum carbon dioxide partial pressure (PCO2), 24-hour carbon dioxide partial pressure change, maximum base excess, maximum white blood cell count, maximum platelet count, minimum red blood cell count, mean corpuscular volume, mean corpuscular hemoglobin concentration, bicarbonate (HCO3–), maximum potassium (K+), maximum sodium (Na+), maximum chloride (Cl–), maximum calcium (Ca+), maximum activated partial thromboplastin time, maximum prothrombin time (PT), maximum blood urea nitrogen (BUN), Glasgow Coma Scale score, and SOFA score.

For the prospective cohort, patients suspected of sepsis or diagnosed with sepsis upon ICU admission had 10 mL of urine collected. The sample was then centrifuged at 3000 rpm for 5 minutes and left to stand for 30 minutes before the supernatant was collected and stored at ≤–70 °C for subsequent analysis. Urinary CCL14 levels were measured using an ELISA kit (Conlon Bioproducts; detection instrument: Rayto RT-6100 microplate reader). Operators were blinded to the experimental research content and strictly adhered to the instructions provided with the kit.

### Model Development and Comparison

The development cohort originated from MIMIC-IV, and it was randomly divided into an 80% training cohort and a 20% validation cohort (internal validation). Ten-fold cross-validation was used for the 80% training set to ensure generalizability. In addition, an external dataset was used for testing (external validation).

The predictive model was developed using 58 features included in the data. Missing data were handled using multiple imputation [39], and the proportion of missing data for each variable can be found in Multimedia Appendix 1. Eight ML models were used to predict persistent SA-AKI, including artificial neural network (ANN), decision tree (DT), gradient boosting machine (GBM), K-nearest neighbor (KNN), logistic regression, categorical boosting (CatBoost), support vector machine (SVM), and extreme gradient boosting (XGBoost). For optimizing the prediction models, Bayesian optimization combined with manual fine-tuning was employed to obtain the final hyperparameters.

To assess the reliability of these models, several commonly used evaluation metrics were adopted, such as area under the receiver operating characteristic curve (AUC), sensitivity, specificity, positive predictive value, negative predictive value, accuracy, and *F*_1_-score.

### Feature Selection and Model Explanation

Interpreting ML models correctly often poses challenges. The SHAP method is a technique that can rank the importance of input features and explain the results of predictive models, and it is commonly used to overcome the “black box” problem in ML [39]. By using SHAP values to assist in feature selection, we reduced the number of features in the predictive model from 58 to 3 based on their importance ranking. We then selected for further analysis the final model with optimal predictive ability during this reduction process. Using the DeLong nonparametric method, we compared differences between AUCs and gradually decreased the number of features in our chosen ML model until there was a significant drop in the AUC.

The SHAP method provides both global and local interpretations for models. Global interpretation offers consistent and accurate attribution values for each feature within a model, demonstrating correlations between input features and persistent SA-AKI. Local interpretation allows specific predictions for individual populations by entering particular data.

### Web Page Deployment Tool Based on the Streamlit Framework

To facilitate the application of this model in a clinical setting, the final prediction model was implemented into a web application built on a Python framework based on Streamlit. When provided with corresponding feature values in the final model, the application can return the probability of persistent SA-AKI.

### Statistical Analysis

Data analysis was conducted using Python 3.9.18 and R 4.2.2 (R Project). Continuous variables with a nonnormal distribution were represented by the median of the IQR and compared using the Mann-Whitney *U* test or Kruskal-Wallis *H* test. Categorical variables were expressed in percentages and compared using the chi-square test or Fisher exact test. Analysis of covariance was used to adjust for confounding factors. We used AUC to assess predictive ability and established optimal cutoff values by maximizing the Youden index (sensitivity + specificity – 1). A 2-tailed *P* value <.05 was considered statistically significant.

## Results

### Patient Characteristics

**Figure 1:**
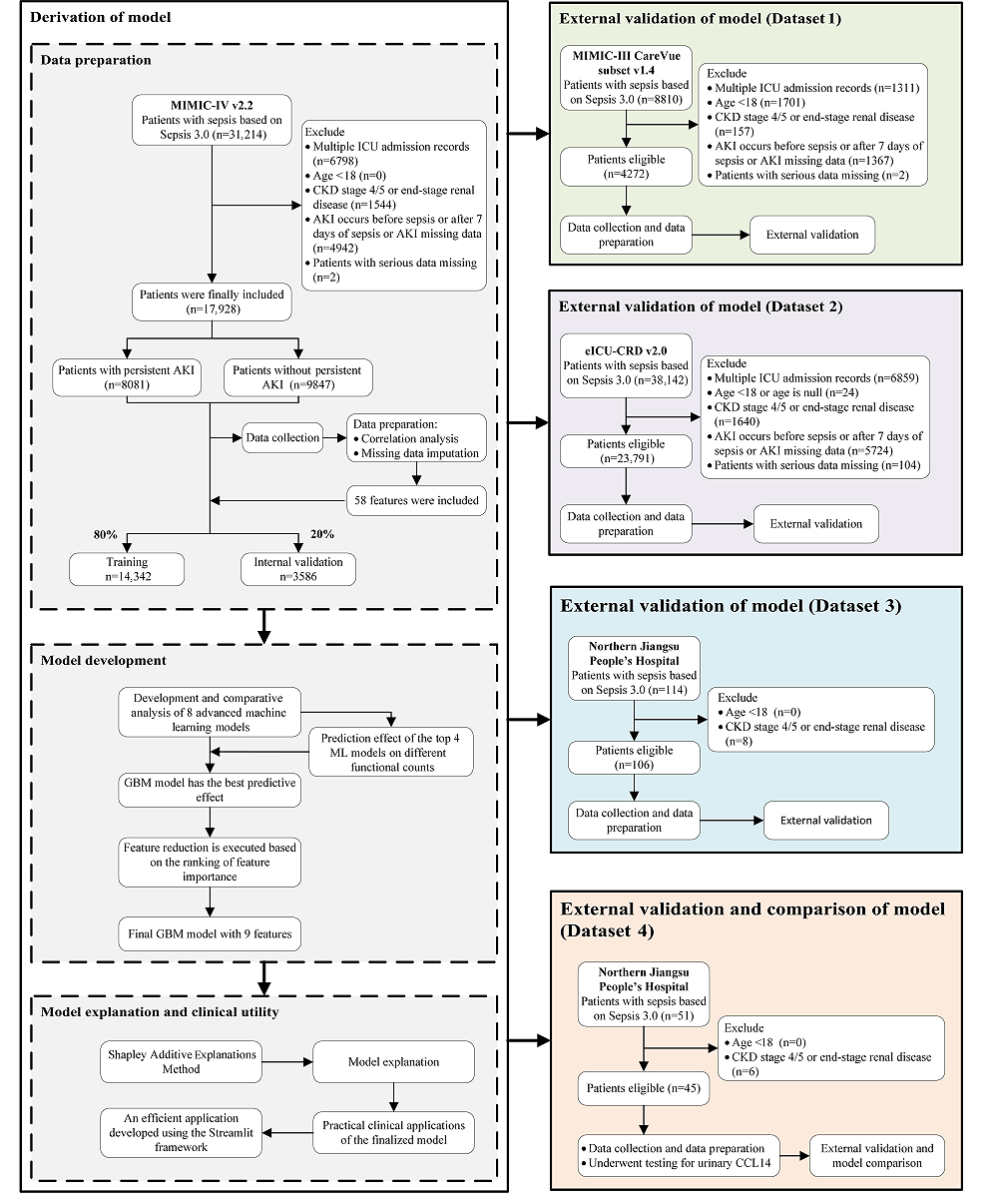
Study flowchart. AKI: acute kidney injury; CCL14: C-C motif chemokine ligand 14; CKD: chronic kidney disease; GBM: gradient boosting machine; ICU: intensive care unit; ML: machine learning; SA-AKI: sepsis-associated acute kidney injury; SHAP: Shapley Additive Explanations.

A total of 17,928 patients with sepsis were included in the MIMIC-IV cohort for predictive model construction. According to the Sepsis-3 criteria, a total of 31,214 patients with sepsis were included in the MIMIC-IV dataset. After excluding 6798 cases with repeated ICU admissions, 1544 cases of stage 4-5 CKD, 4972 cases where AKI occurred before or after 7 days of sepsis onset, and 2 cases of severe data absence, a total of 17,928 patients were analyzed, including those with persistent SA-AKI (n=8081) and those with nonpersistent SA-AKI (n=9847). Out of these patients, 14,342 (80%) were allocated to the validation cohort, while the remaining 3568 (20%) were allocated to the internal validation set. Moreover, the external validation cohort dataset, including the MIMIC-III subset and the e-ICU dataset, used the same exclusion criteria as the MIMIC-IV dataset, with 4272 and 23,791 patients, respectively, being enrolled after the exclusion of patients meeting the exclusion criteria. The Northern Jiangsu People’s Hospital retrospective cohort included 114 patients with sepsis, out of which 8 patients with stage 4-5 CKD were excluded, leaving 106 patients. Furthermore, a prospective cohort at Northern Jiangsu People’s Hospital collected 51 cases, out of which 6 patients with stage 4-5 CKD were excluded, resulting in 45 cases for analysis. Comparisons between the training set, internal verification set, and external verification set regarding demographic statistics and clinical variables can be seen in Multimedia Appendix 3. Detailed information about the research design is available in Figure 1.

Out of the 17,928 patients with sepsis in the derived cohort, 8081 (45.1%) showed progression to persistent SA-AKI, including 2299 who met the AKI stage 1 criteria, 4545 who met the AKI stage 2 criteria, and 1237 who met the AKI stage 3 criteria. Comparison of the demographic and clinical characteristics between patients with nonpersistent SA-AKI and those with persistent SA-AKI can be found in Table 1.

**Table 1:** Comparison of the demographic and clinical characteristics and outcomes between patients with persistent sepsis-associated acute kidney injury and those with nonpersistent sepsis-associated acute kidney injury.

Variable		Nonpersistent SA-AKI^a^ (N=9847)	Persistent SA-AKI (N=8081)	Overall *P*
**AKI^b^ >stage, n (%)**				<.001
	0	5421 (55.1)	0 (0.0)	
	1	2161 (21.9)	2299 (28.4)	
	2	2117 (21.5)	4545 (56.2)	
	3	148 (1.5)	1237 (15.3)	
BMI (kg/m^2^), median (IQR)		26.6 (23.2 to 30.5)	28.9 (25.0 to 34.0)	<.001
Gender (male), n (%)		5658 (57.5)	4741 (58.7)	.11
**Comorbidities, n (%)**				
	Hypertension	4363 (44.3)	3873 (47.9)	<.001
	Diabetes	2547 (25.9)	2486 (30.8)	<.001
	Coronary atherosclerosis	2598 (26.4)	2864 (35.4)	<.001
	Chronic kidney disease	1307 (13.3)	1398 (17.3)	<.001
	Chronic heart failure	2014 (20.5)	2650 (32.8)	<.001
	Chronic liver disease	815 (8.3)	851 (10.5)	<.001
	COPD^c^	1354 (13.8)	1351 (16.7)	<.001
**Infection sources, n (%)**				
	Lung infection	3575 (36.3)	3487 (43.2)	<.001
	Intestinal infection	1284 (13.0)	1078 (13.3)	.57
	Catheter-related infection	475 (4.8)	688 (8.5)	<.001
	Urinary system infection	1814 (18.4)	1507 (18.6)	.71
	Skin infection	468 (4.8)	375 (4.6)	.75
**Severity scale, median (IQR)**				
	GCS^d^ score	15.0 (13.0 to 15.0)	15.0 (13.0 to 15.0)	<.001
	SOFA^e^ score	4.00 (3.00 to 6.00)	6.00 (4.00 to 9.00)	<.001
**Vital signs, median (IQR)**				
	HR^f^_max (beats/min)	103.0 (91.0 to 117.0)	104.0 (91.0 to 119.0)	.01
	MAP^g^_min (mmHg)	59.0 (52.0 to 65.0)	57.0 (50.0 to 62.0)	<.001
	RR^h^_max (times/min)	27.0 (24.0 to 31.0)	28.0 (24.0 to 32.0)	<.001
	Temperature_max (°C)	37.4 (37.0 to 37.9)	37.4 (37.0 to 37.9)	.01
**Laboratory findings, median (IQR)**				
	PaO_2__min (mmHg)	84.8 (56.0 to 109.0)	75.0 (46.0 to 100.0)	<.001
	ΔPaO_2_ (mmHg)	–56.00 (–128.42 to –10.98)	–42.00 (–146.00 to 5.00)	<.001
	PCO_2__max (mmHg)	44.8 (40.0 to 50.0)	47.0 (42.0 to 54.0)	<.001
	ΔPCO_2_ (mmHg)	–0.57 (–2.43 to 1.21)	–1.00 (–4.77 to 2.00)	<.001
	PH^i^_min	7.34 (7.29 to 7.38)	7.32 (7.25 to 7.37)	<.001
	Lactate_max (mmol/L)	2.01 (1.40 to 2.90)	2.40 (1.60 to 3.60)	<.001
	Lactate clearance rate	0.10 (0.01 to 0.19)	0.08 (–0.03 to 0.22)	<.001
	BE^j^_max (mmol/L)	0.00 (–1.00 to 2.00)	0.13 (–1.00 to 3.00)	.003
	Glucose_max (mg/dL)	160.0 (127.0 to 202.0)	173.0 (140.0 to 220.0)	<.001
	BUN^k^_max (mmol/L)	19.0 (13.0 to 29.0)	23.0 (16.0 to 34.0)	<.001
	WBC^l^_max (k/μL)	13.3 (9.5 to 17.8)	14.3 (10.3 to 19.4)	<.001
	Platelets_max (k/μL)	200.0 (146.0 to 270.0)	197.0 (144.0 to 267.0)	.04
	RBC^m^_min (k/μL)	3.33 (2.85 to 3.82)	3.23 (2.76 to 3.78)	<.001
	MCHC^n^_min (g/dL)	32.9 (31.8 to 33.9)	32.5 (31.4 to 33.6)	<.001
	MCV^o^_min (fL)	90.0 (86.0 to 94.0)	90.0 (86.0 to 94.0)	<.001
	RDW^p^_max (%)	14.5 (13.5 to 15.9)	14.8 (13.8 to 16.5)	<.001
	Calcium_max (mEq/L)	8.40 (8.00 to 8.90)	8.45 (8.10 to 8.90)	.002
	Potassium_max (mEq/L)	4.40 (4.00 to 4.80)	4.50 (4.10 to 5.00)	<.001
	Chloride_max (mEq/L)	107.0 (104.0 to 111.0)	107.0 (103.0 to 111.0)	.22
	Anion gap_max (mmol/L)	15.0 (13.0 to 18.0)	16.0 (13.0 to 19.0)	<.001
	PT^q^_max (seconds)	14.5 (12.9 to 16.7)	15.5 (13.5 to 18.9)	<.001
	APTT^r^_max (seconds)	31.9 (28.0 to 38.4)	34.6 (29.4 to 46.6)	<.001
**Intervention**				
	KRT^s^, n (%)	48 (0.5)	522 (6.5)	<.001
	MV^t^, n (%)	4492 (45.6)	5263 (65.1)	<.001
	Norepinephrine, n (%)	1806 (18.3)	2406 (29.8)	<.001
	Diuretic, n (%)	2556 (26.0)	3053 (37.8)	<.001
	Furosemide dose (mg), median (IQR)	0.00 (0.00 to 0.00)	0.00 (0.00 to 20.00)	<.001
	Statin, n (%)	3258 (33.1)	3166 (39.2)	<.001
	ACEI/ARB^u^, n (%)	866 (8.8)	758 (9.4)	.18
	Aminoglycoside, n (%)	275 (2.8)	252 (3.1)	.22
	Glycopeptide, n (%)	5355 (54.4)	4672 (57.8)	<.001
	NSAID^v^, n (%)	3643 (37.0)	3710 (45.9)	<.001
	Acyclovir, n (%)	457 (4.6)	264 (3.3)	<.001
	Fluid balance, median (IQR)	2333 (470 to 4744)	3547 (1131 to 6362)	<.001
	Urine output, median (IQR)	1910 (1300 to 2754)	1345 (805 to 2015)	<.001
**Outcome**				
	Length of stay (days), median (IQR)	1.99 (1.23 to 3.26)	3.79 (1.86 to 7.18)	<.001
	ICU^w^ mortality, n (%)	277 (2.8)	1326 (16.4)	<.001

^a^SA-AKI: sepsis-associated acute kidney injury.

^b^AKI: acute kidney injury.

^c^COPD: chronic obstructive pulmonary disease.

^d^GCS: Glasgow Coma Scale.

^e^SOFA: Sequential Organ Failure Assessment.

^f^HR: heart rate.

^g^MAP: mean arterial pressure.

^h^RR: respiratory rate.

^i^PH: potential of hydrogen.

^j^BE: base excess.

^k^BUN: blood urea nitrogen.

^l^WBC: white blood cell.

^m^RBC: red blood cell.

^n^MCHC: mean corpuscular hemoglobin concentration.

^o^MCV: mean corpuscular volume.

^p^RDW: red blood cell distribution width.

^q^PT: prothrombin time.

^r^APTT: activated partial thromboplastin time.

^s^KRT: kidney replacement therapy.

^t^MV: mechanical ventilation.

^u^ACEI/ARB: angiotensin-converting enzyme inhibitor/angiotensin receptor blocker.

^v^NSAID: nonsteroidal anti-inflammatory drug.

^w^ICU: intensive care unit.

### Model Development and Performance Comparison

**Figure 2:**
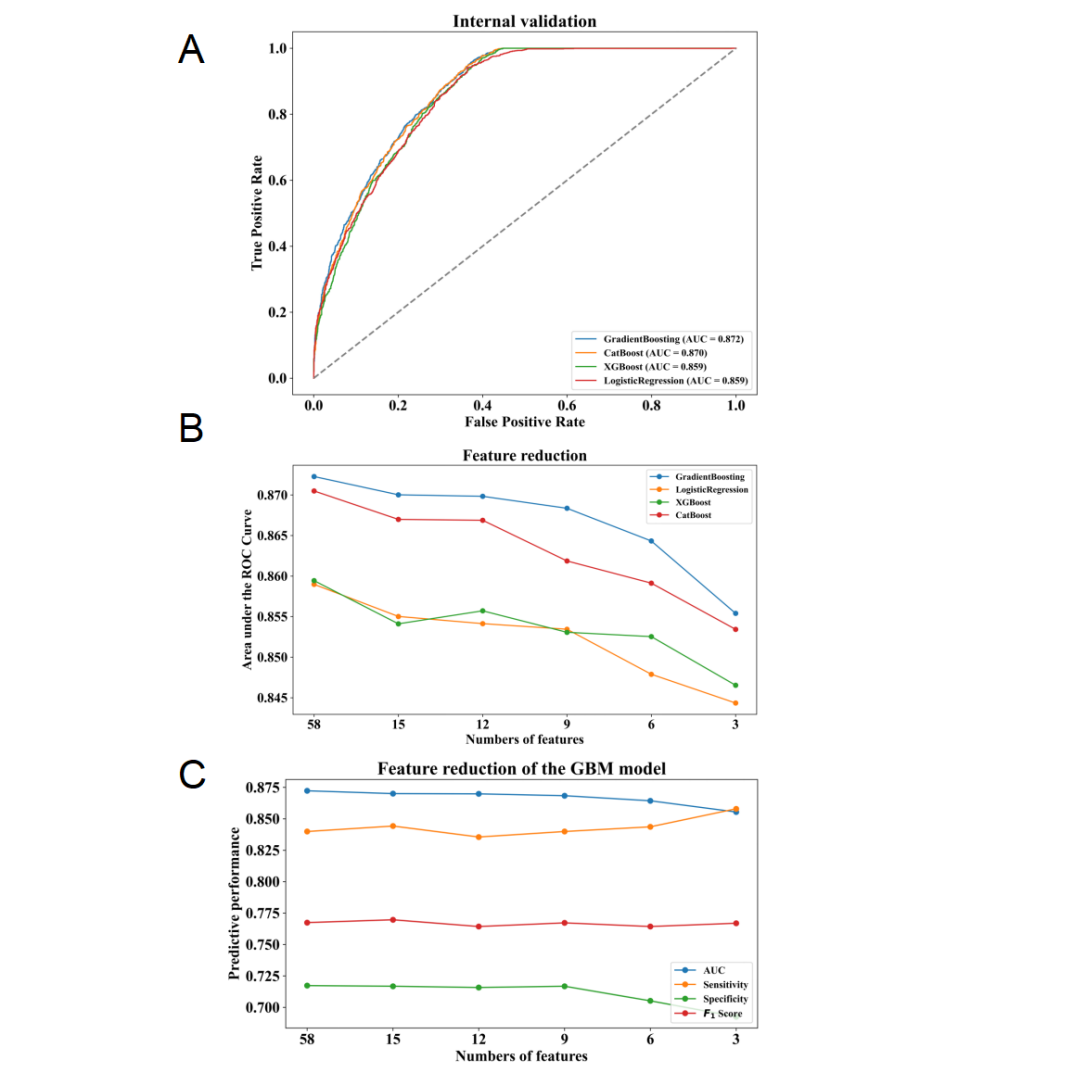
Performance of machine learning (ML) models to predict persistent sepsis-associated acute kidney injury (SA-AKI). (A) Receiver operating characteristic curves of the top 4 best performing ML models. (B) Areas under the curve (AUCs) of the top 4 best performing ML models with varied numbers of features. (C) AUC, sensitivity, specificity, and F1-score of the gradient boosting machine (GBM) model with varied numbers of features. CatBoost: categorical boosting; XGBoost: extreme gradient boosting.

Eight ML models were generated using clinical data collected within 24 hours after admission to the ICU in order to predict whether patients with sepsis would develop persistent SA-AKI during this period. Among these 8 models, the GBM model (AUC=0.872) had the best predictive performance for persistent SA-AKI, followed by the CatBoost model (AUC=0.870) and XGBoost model (AUC=0.859). The discriminative abilities of these 8 models can be seen in Multimedia Appendix 4, and the receiver operating characteristic curves and SHAP summary plots for the top 12 features of the 4 best performing ML models are shown in Figure 2A and Multimedia Appendix 5, respectively.

During feature reduction based on feature importance rankings, changes in AUC among the 4 types of models indicated that GBM maintained almost optimal predictive ability among all 4 types of models (Figure 2B). Therefore, it was observed that among the aforementioned models, GBM performed the best at predicting persistent SA-AKI. The performance of GBM with different numbers of features is illustrated in Figure 2C and Multimedia Appendix 6.

### Identification of the Final Model

The final model was determined during the feature reduction process of the GBM model. As shown in Figure 2C and Multimedia Appendix 7, the 58-feature model significantly outperformed the 3-feature model (ΔAUC=0.017; *P*<.001), 6-feature model (ΔAUC=0.008; *P*<.001), and 9-feature model (ΔAUC=0.004; *P*=.03) in predicting whether ICU patients would develop persistent SA-AKI, but it did not have a significant advantage over the 12-feature model (ΔAUC=0.002; *P*=.13). Compared with the 58-feature model, the 12-feature model showed better net benefits at higher threshold probabilities. Meanwhile, its area under the precision-recall curve was only slightly lower than that of the 58-feature model, indicating that both models had similar and high clinical utility (Multimedia Appendix 7). Therefore, we chose to use a GBM model based on AKI stage, ΔCreatinine, urine output, furosemide dose, BMI, SOFA score, KRT, mechanical ventilation, BUN, lactate, PT, and age for further analysis. Finally, the AUC of our ultimate GBM model for predicting persistent SA-AKI was found to be at an impressive level of 0.870 (95% CI 0.859-0.870), with a sensitivity of 0.836, specificity of 0.716, positive predictive value of 0.704, negative predictive value of 0.843, accuracy of 0.769, and *F*_1_-score of 0.764. In addition, further cross-validation was performed to verify the appropriate sample size for this study and the robustness of the model to locus variation. As shown in Multimedia Appendix 8, the final model had mean AUCs of 0.883 (SD 0.009) and 0.884 (SD 0.008) for 5-fold and 10-fold cross-validation, respectively.

### External Validation of the Final Model

**Figure 3:**
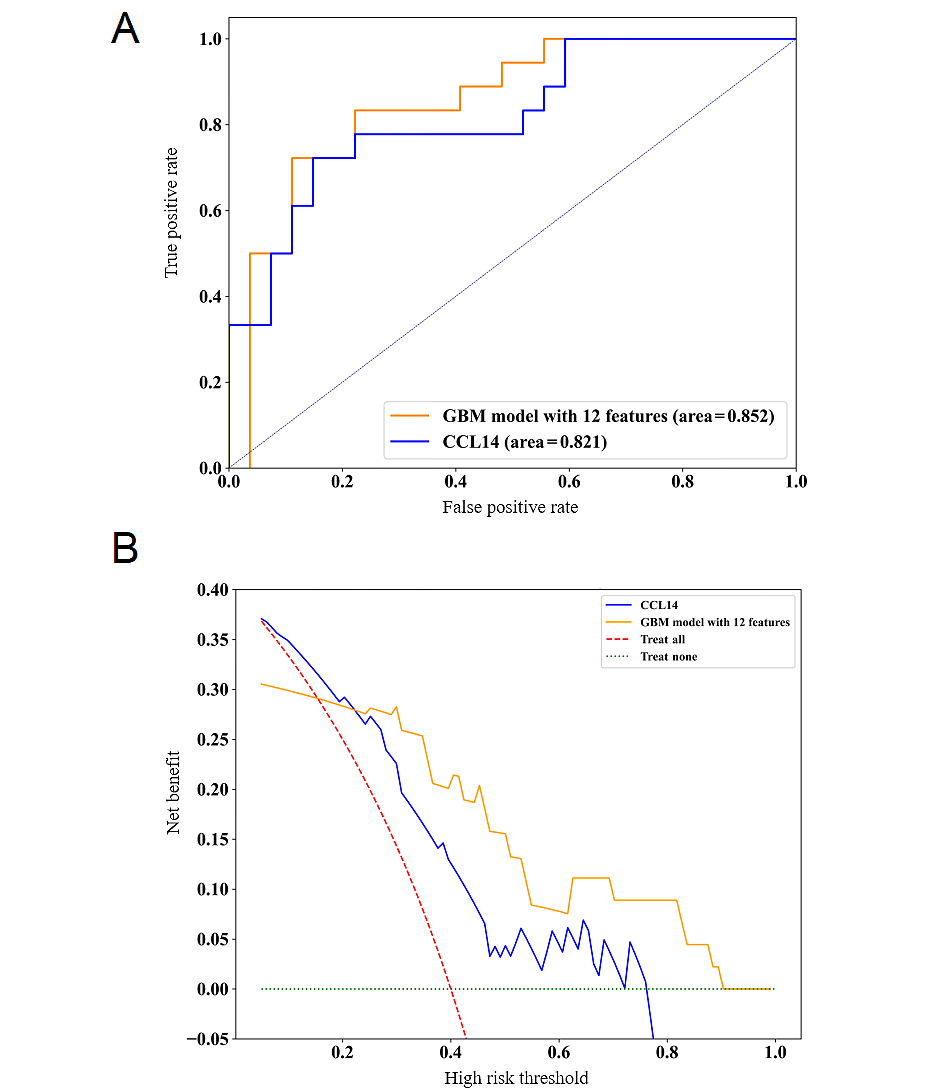
Comparison between the final gradient boosting machine (GBM) model and C-C motif chemokine ligand 14 (CCL14). (A, B) Receiver operating characteristic curves (A) and decision curve analysis curves (B) of CCL14 and the final GBM model with 8 features. These plots represent the predictive performance in the prospective external validation cohort.

For external validation, the final model achieved an AUC of 0.891 on a subset of the MIMIC-III dataset, an AUC of 0.932 on the e-ICU dataset, and an AUC of 0.983 for a retrospective cohort from Northern Jiangsu People’s Hospital. These results are similar to those obtained during internal validation (AUC=0.870) (Table 2), indicating that our final model demonstrated excellent diagnostic performance in both internal and external validations. We further compared our final model with a biomarker known for its high accuracy in predicting persistent SA-AKI within a prospective cohort study. The results suggest that the AUC of our model was higher than that of CCL14 (ΔAUC=0.031) (Figure 3A). The decision curve analysis curve also showed that our final model had greater clinical utility compared to CCL14 (Figure 3B).

**Table 2:** Performance of the gradient boosting machine model with different external validations for persistent sepsis-associated acute kidney injury prediction.

Dataset	AUROC^a^	AUPRC^b^	Accuracy	*F*_1_-score	Precision	Recall	Specificity	NPV^c^	Youden index
e-ICU^d^	0.932	0.795	0.850	0.722	0.692	0.754	0.884	0.912	0.637
Retrospective cohort of Northern Jiangsu People’s Hospital	0.983	0.981	0.909	0.909	0.833	1.000	0.833	1.000	0.833
Prospective cohort of Northern Jiangsu People’s Hospital	0.852	0.742	0.822	0.765	0.813	0.722	0.889	0.828	0.611
MIMIC-III	0.891	0.848	0.802	0.794	0.744	0.851	0.763	0.863	0.614

^a^AUROC: area under the receiver operating characteristic curve.

^b^AUPRC: area under the precision-recall curve.

^c^NPV: negative predictive value.

^d^ICU: intensive care unit.

### Model Explanation

**Figure 4:**
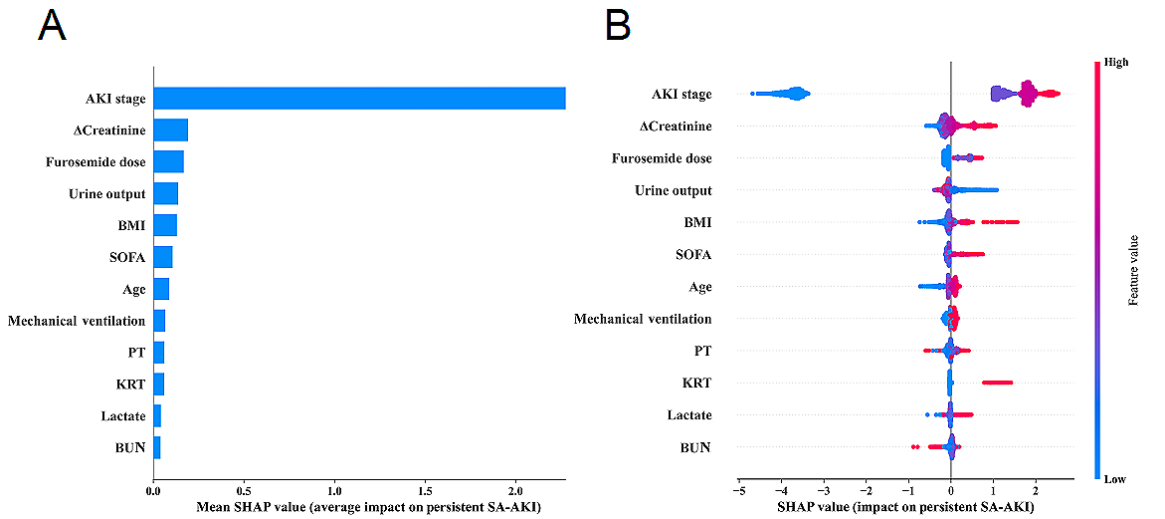
Global model explanation by the Shapley Additive Explanations (SHAP) method. (A) SHAP summary bar plot. (B) SHAP summary dot plot. The probability of persistent SA-AKI development increases with the SHAP value of a feature. A dot is made for a SHAP value in the model for each patient, and thus, each patient has 1 dot on the line for each feature. The colors of the dots demonstrate the actual values of the features for each patient (red indicates a higher feature value, and blue indicates a lower feature value). The dots are stacked vertically to show density. AKI: acute kidney injury; BUN: blood urea nitrogen; ΔCreatinine: changes in creatinine within 24 hours; KRT: kidney replacement therapy; PT: prothrombin time; SA-AKI: sepsis-associated acute kidney injury; SOFA: Sequential Organ Failure Assessment.

**Figure 5:**
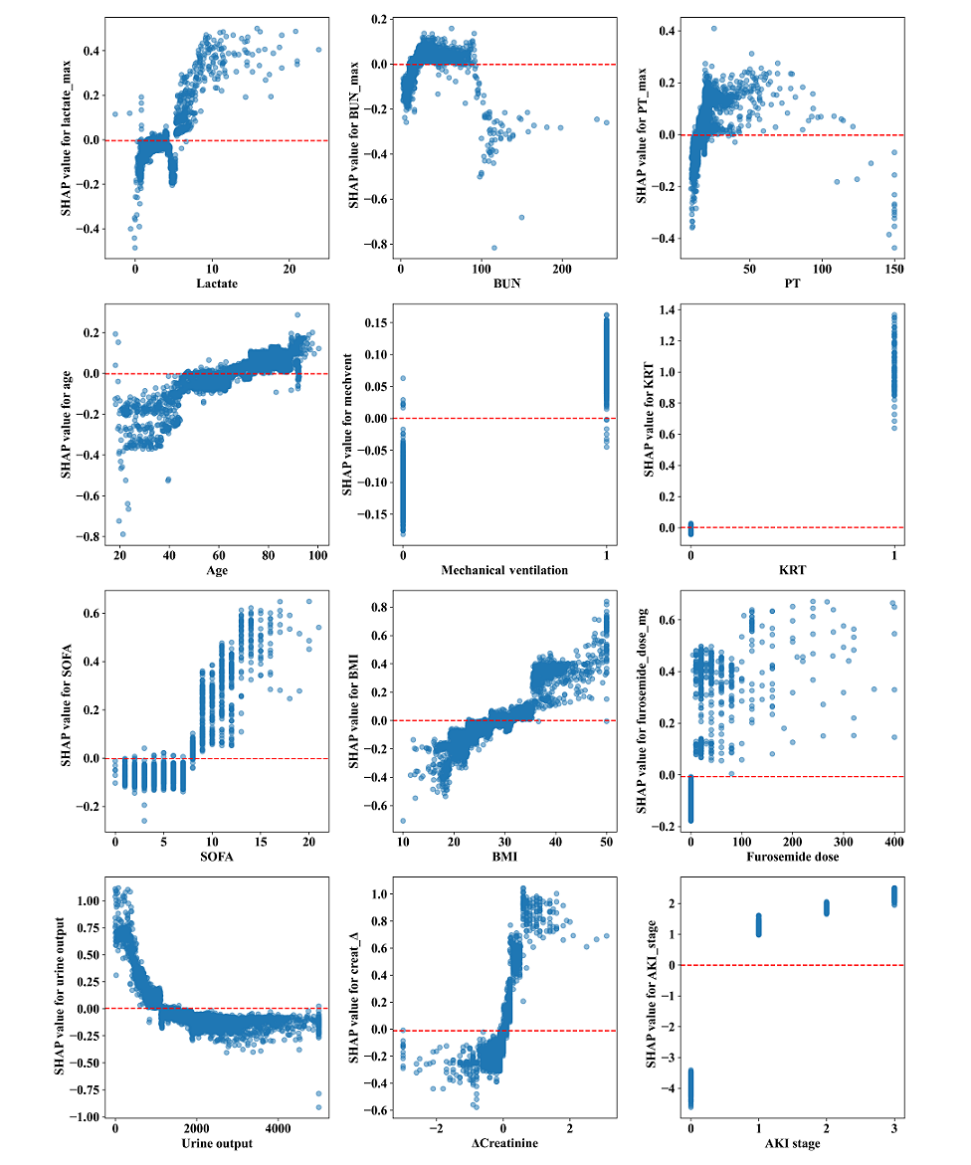
Shapley Additive Explanations (SHAP) dependence plot. Each dependence plot shows how a single feature affects the output of the prediction model, and each dot represents a single patient. For example, Sequential Organ Failure Assessment (SOFA) scores above 8 points, urine output less than 1380 mL/24 h, BMI above 24.92 kg/m^2^, or age older than 66 years push the decision toward the “persistent sepsis-associated acute kidney injury (SA-AKI)” class. SHAP values are represented by the y-axis, and actual values are represented by the x-axis. SHAP values for specific features exceeding zero push the decision toward the “persistent SA-AKI” class. AKI: acute kidney injury; BUN: blood urea nitrogen; ΔCreatinine: changes in creatinine within 24 hours; KRT: kidney replacement therapy; PT: prothrombin time.

Given that clinicians often struggle to accept a predictive model they cannot directly interpret and understand, we employed the SHAP method to explain the output of our final model. This was achieved by calculating each variable’s contribution to the prediction. This interpretable approach provides 2 types of explanations: global interpretation at the feature level and local interpretation at the individual level. Global interpretation describes the overall function of the model. As shown in SHAP summary plots (Figure 4A and 4B), features’ contributions to models were evaluated using average SHAP values and displayed in descending order. Additionally, SHAP dependence plots helped understand how individual features impact predictions from our model. Figure 5 shows a comparison between actual values for the 12 features and their corresponding SHAP values, where a positive value indicates a higher risk for persistent SA-AKI within our model, or put differently, it pushes decisions toward “persistent SA-AKI.” For instance, AKI stages of ≥1, creatinine changes over 24 hours of ≥0.1 mg/dL, use of furosemide on admission day, KRT, and presence of sepsis along with KRT all have positive SHAP values that push decisions toward “persistent SA-AKI.” Similarly, SOFA scores above 8 points, urine output of <1380 mL/24 h, BMI of >24.92 kg/m^2^, and age older than 66 years push decisions toward this category.

**Figure 6:**
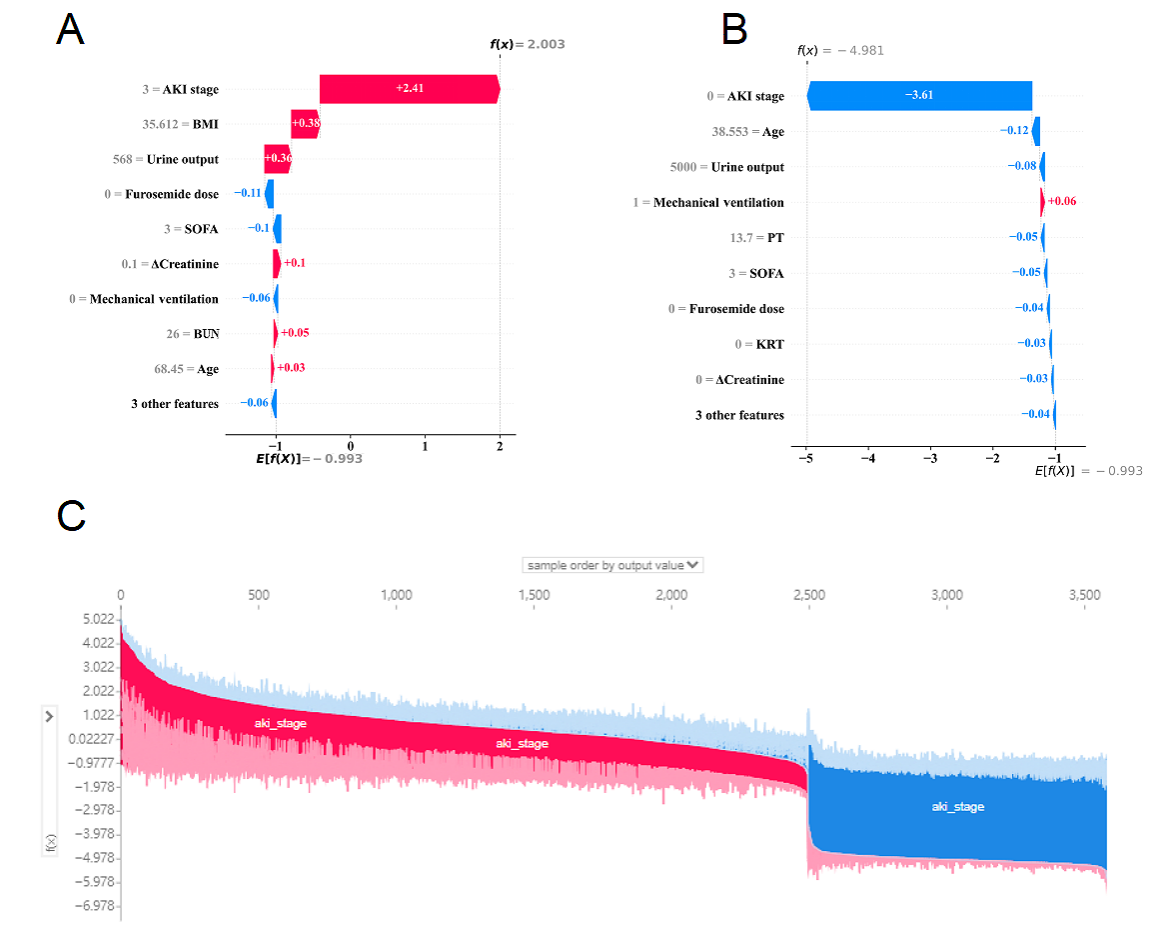
Local model explanation by the Shapley Additive Explanations (SHAP) method. (A) Patient with sepsis in the “persistent sepsis-associated acute kidney injury (SA-AKI)” class. (B) Patient with sepsis in the “nonpersistent SA-AKI” class. (C) Force plot for the internal validation set. Each patient is represented by the x-axis, while the feature contribution is represented by the y-axis: an increased red part for each individual patient represents a greater probability toward the decision of “persistent SA-AKI.” AKI: acute kidney injury; BUN: blood urea nitrogen; ΔCreatinine: changes in creatinine within 24 hours; KRT: kidney replacement therapy; PT: prothrombin time; SOFA: Sequential Organ Failure Assessment.

Furthermore, local interpretation analyzes how specific predictions are made for particular individuals, including personalized input data. Figure 6A presents the data of a patient who developed persistent SA-AKI during their ICU stay. According to our predictive model, this patient was categorized under “persistent SA-AKI,” with a probability of 87%. The figure shows that factors, such as AKI stage, BMI, urine output, ΔCreatinine, and age, pushed decision-making toward persistent SA-AKI, while lower SOFA scores, absence of mechanical ventilation, KRT, and diuretics reduced the risk of AKI. Conversely, these factors could increase the patient’s risk for persistent SA-AKI.

Figure 6B presents the data of a patient who did not develop persistent SA-AKI during their ICU stay. Figure 6B shows the features and their actual measurements that pushed decision-making toward “nonpersistent SA-AKI.” The decision in this case leaned toward “nonpersistent SA-AKI,” with a probability of 0.47%. Additionally, Figure 6C shows an explanatory power chart for patients in our internal validation cohort. The x-axis represents each patient, while the y-axis indicates feature contributions. Each individual patient shows increased red portions, indicating a higher probability for a “persistent SA-AKI” decision.

### Convenient Application for Clinical Utility

**Figure 7:**
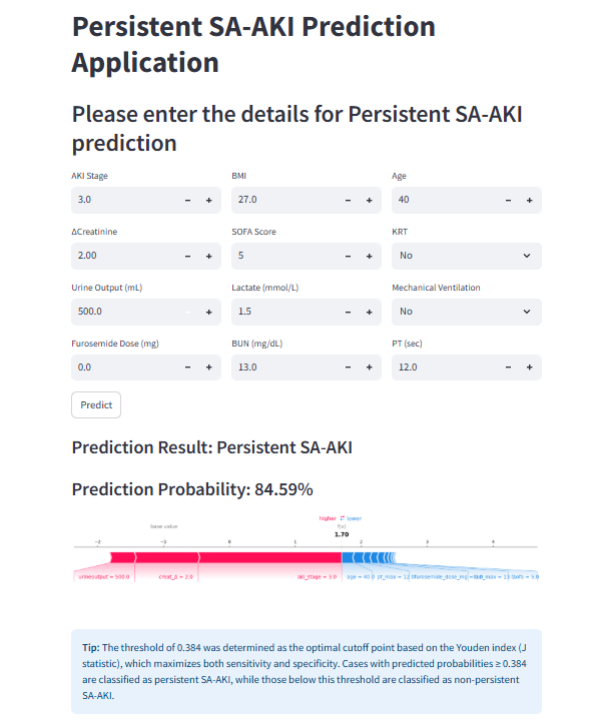
Convenient application for clinical utility. The convenient application of the final gradient boosting machine (GBM) model with 9 features is available for persistent sepsis-associated acute kidney injury (SA-AKI) prediction. For example, after entering the actual values of the 9 features, this application automatically displays a probability of 82.35%. The force plot indicates the features that contribute to the decision of “persistent SA-AKI.” The blue features on the right are the features pushing the prediction toward the “nonpersistent SA-AKI” class, while the red features on the left are the features pushing the prediction toward the “persistent SA-AKI” class.

For the convenience of clinical application, the final prediction model was implemented into a web-based application (Figure 7). When the actual values of the 9 features required by the model are inputted, this application automatically predicts an individual patient’s risk of persistent SA-AKI. In addition, it displays an interpretive force plot for each patient to indicate which features contribute to decisions about persistent SA-AKI: blue features on the right push predictions toward “nonpersistent SA-AKI,” while red features on the left push predictions toward “persistent SA-AKI.” This web application can be accessed online [40]. The code for building the web version of the application is provided in Multimedia Appendix 9.

## Discussion

### Principal Findings

This study not only established a model of persistent SA-AKI using a large database involving a cohort of patients with sepsis, but also validated the model with retrospective and prospective cohorts. The model uses routine clinical data to predict persistent SA-AKI in patients with sepsis in the ICU environment quite well, and its diagnostic performance is even superior to that of CCL14, a biomarker known for diagnosing persistent SA-AKI.

Persistent SA-AKI has poor clinical prognosis, and thus, early identification and intervention are crucial. However, a single-method diagnosis has limited effectiveness, including traditional urine indicators, renal ultrasound, and biomarkers [10-19]. ML is an appealing method for predicting persistent SA-AKI. ML technology is a powerful computational method for handling complex and extensive data, as it can manage highly variable datasets while understanding complex relationships between variables in a flexible and trainable manner. EMR data are easily accessible and accurate clinical data for clinicians and researchers. When combined with sophisticated ML algorithms, they can promote the development of clinical prediction models [41]. In this study, among 8 ML models tested, the GBM model had the best AUC, demonstrating good net benefits along with higher feature reduction threshold probabilities. GBM consists of many simple DTs, making it highly interpretable. Multiple studies have proven that GBM methods hold great predictive value within medical fields [42-44]. In our research, we used GBM to develop our final model featuring 9 characteristics that can be easily obtained or evaluated.

The number of features for any given model remains elusive due to a lack of guidelines or consensus on selecting features for predictive models. While more features may provide additional information for predictive models, including numerous features could limit their clinical application, and incorporating noncausal features might reduce prediction accuracy [45]. The use of SHAP methodology assisted in feature selection, resulting in our final simplified yet convenient ML predictive model, which can be easily applied to facilitate clinical decision-making for patients with sepsis.

This study attempted to apply ML algorithms to predict the occurrence of persistent AKI in patients with sepsis. In our complex GBM model, we found that factors, such as AKI stage, creatinine change value, urine output, diuretic dosage, mechanical ventilation, KRT, BMI, SOFA score, BUN, lactate, PT, and age, are associated with the development of persistent SA-AKI. Previous studies have shown a positive correlation between the severity of AKI in patients with this subtype and persistent AKI. Compared to transient AKI, patients with persistent AKI have higher AKI stages and SOFA scores [46,47]. The presence of a higher AKI stage and SOFA score is indicative of more severe kidney injury. As the severity of the injury escalates, the structural and functional impairments of the kidney are exacerbated, leading to an increase in the number of damaged renal units. The self-repairing and compensatory capacity of the kidney gradually diminishes, increasing the likelihood of developing persistent AKI. A higher stage is frequently accompanied by more severe inflammatory reactions and alterations in renal hemodynamics, providing a pathological foundation for the progression of persistent kidney injury. Our findings align with those of previous studies [46,47]. The SHAP values from our GBM model suggest that higher AKI stages and higher SOFA scores contribute to the occurrence of persistent SA-AKI. Urine output is considered one of the most important indicators for diagnosing AKI, which is consistent with the KDIGO recommendations. Oliguria is a significant risk factor for developing persistent SA-AKI, and decreased urine output is indicative of inadequate renal perfusion or renal tubular dysfunction, which in turn results in decreased urine production. Inadequate renal perfusion results in oxygen deprivation of renal tissues, triggering cell damage and apoptosis. In contrast, renal tubular dysfunction affects the concentration and excretion of urine, thereby preventing the timely excretion of metabolites, which accumulate in the body and cause further damage to the kidneys, increasing the risk of persistent AKI. However, there remains controversy over whether using diuretics has any association with preventing or treating AKI [48-51]. Theoretically speaking, diuretics can prevent AKI by reducing GFR and tubular reabsorption while also decreasing medullary oxygenation [52-54]. Some researchers believe that diuretics can act as renal vasodilators, thereby preventing AKI [55]. However, contrary to this belief, we found through our research that the use of diuretics plays an important role in influencing outcomes related to AKI. Consistent with our findings, several newly published studies [56] have discovered increased risks associated with the use of diuretics, leading to AKI. A meta-analysis conducted by Ho and Sheridan [56] involving 9 different studies showed that diuretics have no significant clinical benefits in preventing or treating AKI in adults. Worse still, treatment with diuretics may prolong hospital stay, and high doses of diuretics carry a short-term risk of deafness or tinnitus, which is harmful for ICU patients [56]. Therefore, renal function must be monitored during the course of treatment with diuretics. Creatinine change as a core indicator of the renal angina index (RAI) has been proven to predict the occurrence of AKI and persistent AKI in both adults and children [57-59]. The RAI is determined by subtle changes in the patient’s condition and kidney function. It represents a special concept in patients at high risk for persistent AKI. Our study suggested that creatinine changes within 24 hours are associated with persistent SA-AKI. It is worth noting that mechanical ventilation is significantly associated with persistent SA-AKI, and a significant increase in creatinine indicates substantial impairment of the kidney filtration function, potentially resulting from renal parenchyma damage and a decline in the GFR. The inability of the kidneys to effectively remove creatinine leads to the accumulation of metabolic waste products within the body, which in turn can result in further damage to kidney cells, the triggering of an inflammatory response and oxidative stress, and the exacerbation of AKI. Positive pressure ventilation is commonly used to provide oxygenation, ventilation, and airway protection support for critically ill patients. However, positive pressure ventilation has long been considered potentially harmful to the kidneys [60]. This could be due to 3 reasons. First, positive pressure ventilation might increase intrathoracic pressure, thereby reducing venous return, cardiac output, and renal perfusion. Second, mechanical ventilation can induce the release of certain neurohormones that affect the renin-angiotensin system, leading to reduced renal blood flow and estimated GFR. Third, any form of mechanical ventilation under any volume or pressure can trigger cascade inflammation involving multiple leukocyte interleukins, tumor necrosis factor-α, and the Fas ligand, which might lead to AKI. Aging and obesity are common risk factors for AKI. Obesity leads to glomerular hypoperfusion, which can increase single-nephron hemodynamics and metabolic burden and activate adipocyte inflammation and oxidative stress, thus increasing the risks associated with the progression to AKI [7]. In sepsis, the inflammatory response activates the coagulation system, leading to extensive microthrombosis (eg, disseminated intravascular coagulation) and depletion of coagulation factors and platelets, which ultimately manifests as prolonged PT. Prolonged PT is often an indicator of sepsis severity and poor prognosis, and correlates with persistent SA-AKI and increased mortality. Persistent elevation of lactate is an indicator of poor prognosis. In sepsis, microcirculatory dysfunction leads to tissue hypoxia, anaerobic metabolism produces lactate, and hyperlactatemia further exacerbates acidosis and renal tubular injury, leading to the development of persistent AKI. With advancing age, the kidney undergoes a series of structural and functional changes, including a decrease in the number of renal units, glomerulosclerosis, tubular atrophy, and a decline in reserve function and compensatory capacity. Consequently, when subjected to identical injury factors, the kidneys of elderly individuals are more susceptible to AKI. Due to their diminished repair capacity, recovery is more challenging in these individuals, and there is an increased risk of persistent AKI. Since these indicators are easy to assess at the time of admission, they can serve as convenient predictive factors for critically ill patients with sepsis who might develop persistent SA-AKI. In summary, the majority of the model’s key features, including AKI classification, creatinine change values, urine output, mechanical ventilation, KRT, age, BMI, BUN, lactate, PT, and SOFA scores, are consistent with clinical risk and contribute to the worsening of AKI. However, our study also identified some discrepancies that are of concern. For instance, the established correlation between diuretic dosage and AKI recovery deviated from conventional wisdom. Higher doses of diuretics did not promote recovery of renal function as expected and were associated with a poor prognosis to some extent. This finding calls into question the conventional wisdom surrounding the effectiveness of diuretics in the treatment of AKI and suggests that clinicians should exercise greater caution when prescribing diuretics.

### Comparison With Prior Work

As previously reported, some studies have established AKI prediction models based on ML methods [21-26], which have focused on unsubtyped AKI. This status quo is not conducive to guiding clinics toward personalized treatments. Only a few studies have used ML models to predict persistent AKI [27,61]. For instance, Ding et al [61] found that serum albumin, CKD, AKI stage, SOFA score, lactate on the first day, and KRT were significantly associated with persistent AKI through least absolute shrinkage and selection operator regression and support vector machine recursive feature elimination analyses using a free database. Using these predictors, a column-line graph was constructed. The diagnostic efficacy of this column chart in predicting persistent AKI in the training set was 0.730, whereas in the external validation cohort, it was only 0.702. It is noteworthy that several of the factors examined in this study, including AKI stage, SOFA score, lactate, and KRT, are similar to those observed in our study. Nevertheless, the predictive model developed in this study demonstrated unsatisfactory diagnostic efficacy in both the training and validation cohorts. Jiang et al [27] conducted a retrospective analysis of 955 patients with postsurgical comorbid AKI and predicted the occurrence of persistent AKI using a model integrated with 3 ML methods. The model demonstrated a diagnostic value of 0.86 in the training set and a comparatively modest diagnostic value of 0.693 in an external validation cohort. This limitation directly restricts the generalizability of the model. In comparison with preceding studies, the constructed model has a number of advantages. First, the model demonstrates remarkable stability, with a diagnostic value for predicting persistent AKI of >0.85 in both the training and validation cohorts, thus demonstrating excellent diagnostic performance. Second, we seek to address the “black box” problem of ML methods that are difficult to interpret directly, by using the SHAP method to profile ML models. The model can be interpreted at both the global and local levels. The global interpretation describes the overall functionality of the model, while the local interpretation makes predictions specific to individual patients with sepsis through the input of personalized data. This facilitates a more profound comprehension of the ML model’s inner workings by clinicians, thereby enhancing their propensity to make informed medical decisions. Third, the prediction models constructed in previous studies are difficult to use in clinical applications. In response to this, an online application was developed based on the Flow Light framework to facilitate clinicians’ access and assist them in clinical decision-making. Fourth, there is a paucity of studies that have until now compared self-developed models with established markers in prospective cohorts. In this study, an ML model was compared with CCL14, a chemokine belonging to the CC chemokine family that plays an important role in immune regulation and inflammatory responses [19]. It has been established that CCL14 is closely associated with the degree of renal tubular injury and disease progression, and its elevated urinary concentration may reflect the activation of local inflammatory responses in renal tissues, which are involved in the processes of renal tubular epithelial cell apoptosis and interstitial fibrosis. Available studies have demonstrated that CCL14 exhibits good diagnostic efficacy for persistent severe AKI (SA-AKI), with the efficacy of urinary CCL14 alone for the prediction of persistent SA-AKI being above 0.80 [19,62], and thus, it is currently recognized as the most effective predictor of persistent severe AKI [20,63]. Our ML model outperformed CCL14, further highlighting the superiority and reliability of our model.

In summary, we successfully established an interpretable ML model that predicts persistent SA-AKI among patients with sepsis in the ICU environment based on easily extractable clinical data. The final GBM model had good predictive ability in both internal and external validations, and its diagnostic efficacy was superior to that of CCL14 in prospective cohort validation. Further randomized and controlled studies are needed to determine whether individualized and timely treatment measures based on the final prediction model can improve the prognosis of patients with persistent SA-AKI.

### Limitations

This study has some limitations. First, the baseline sCr values for most patients could not be obtained. We used an estimation equation to determine the baseline sCr, and this approach has been employed in many AKI studies to minimize errors as much as possible. Second, we only included information on demographics, vital signs, and laboratory values, which limited the performance of the model and prevented it from discovering more potential factors that contribute to persistent SA-AKI. Lastly, our model only included patients with sepsis. However, AKI is a syndrome with various causes of onset, and thus, it remains unclear whether this model can predict persistent AKI caused by other factors. Further verification is needed in this regard.

### Conclusions

We successfully established an interpretable GBM model for predicting persistent SA-AKI based on readily extractable clinical data. This model demonstrated good predictive ability in both internal and external validation cohorts and outperformed the biomarker CCL14 in prospective cohort validation.

## Data Availability

Data are available upon reasonable request.

## Appendix

Multimedia Appendix 1 Demographic characteristics, vital sign measurements, and laboratory variables with the percentage of missing data.

Multimedia Appendix 2 Heat map of Spearman correlation analyses among variables.

Multimedia Appendix 3 Comparison of demographic and clinical characteristics among the training, internal validation, and external validation cohorts.

Multimedia Appendix 4 Performance of the machine learning models for persistent sepsis-associated acute kidney injury prediction.

Multimedia Appendix 5 Shapley Additive Explanations (SHAP) summary dot plots of the 12 features of the top 4 best performing machine learning models.

Multimedia Appendix 6 Performance of the gradient boosting machine model with varied numbers of features for persistent sepsis-associated acute kidney injury prediction.

Multimedia Appendix 7 Predictive performance of the gradient boosting machine model after reducing features.

Multimedia Appendix 8 Predictive performance of the final gradient boosting machine model in the cross-validation.

Multimedia Appendix 9 Web application codes.

## Abbreviations

ADQI: Acute Disease Quality Initiative
AKI: acute kidney injury
AUC: area under the receiver operating characteristic curve
BUN: blood urea nitrogen
CatBoost: categorical boosting
CCL14: C-C motif chemokine ligand 14
CKD: chronic kidney disease
DT: decision tree
ELISA: enzyme-linked immunosorbent assay
EMR: electronic medical record
GBM: gradient boosting machine
GFR: glomerular filtration rate
ICU: intensive care unit
KDIGO: Kidney Disease: Improving Global Outcomes
KRT: kidney replacement therapy
MIT: Massachusetts Institute of Technology
ML: machine learning
PT: prothrombin time
SA-AKI: sepsis-associated acute kidney injury
sCr: serum creatinine
SHAP: Shapley Additive Explanations
SOFA: Sequential Organ Failure Assessment
XGBoost: extreme gradient boosting
