# Supplementary material for: Explainable Machine Learning Model for Predicting Persistent Sepsis-Associated Acute Kidney Injury: Development and Validation Study
Source: J Med Internet Res. 2025 Apr 28;27:e62932. doi: 10.2196/62932 (PMC12070005; doi:10.2196/62932)
Supplement: Multimedia Appendix 1 [file jmir_v27i1e62932_app1.docx]

**Multimedia Appendix 1.** Demographic characteristics, vital sign measurements, and laboratory variables with the percentage of missing data.

| Variables | e-ICU | Percentage of missing data, % | MIMIC-III | Percentage of missing data, % | MIMIC-IV | Percentage of missing data, % | Northern Jiangsu People’s Hospital | Percentage of missing data, % |
| --- | --- | --- | --- | --- | --- | --- | --- | --- |
| **Persistent AKI n (%)** | 23791 | 0.000 | 4272 | 0.000 | 17928 | 0.000 | 106 | 0.000 |
| AKI stage | 23791 | 0.000 | 4272 | 0.000 | 17928 | 0.000 | 106 | 0.000 |
| **Demographic data** |  |  |  |  |  |  |  |  |
| Age, years | 23791 | 0.000 | 4272 | 0.000 | 17928 | 0.000 | 106 | 0.000 |
| Body weight, kg | 23395 | 0.017 | 3967 | 0.071 | 17743 | 0.010 | 106 | 0.000 |
| Height, cm | 23584 | 0.009 | 1772 | 0.585 | 14740 | 0.178 | 106 | 0.000 |
| Gender, Male, n% | 23788 | 0.000 | 4272 | 0.000 | 17928 | 0.000 | 106 | 0.000 |
| **Comorbidities, n%** |  |  |  |  |  |  |  |  |
| Hypertension | 23791 | 0.000 | 4272 | 0.000 | 17928 | 0.000 | 106 | 0.000 |
| Diabetes | 23791 | 0.000 | 4272 | 0.000 | 17928 | 0.000 | 106 | 0.000 |
| Coronary atherosclerosis | 23791 | 0.000 | 4272 | 0.000 | 17928 | 0.000 | 106 | 0.000 |
| Chronic kidney disease | 23791 | 0.000 | 4272 | 0.000 | 17928 | 0.000 | 106 | 0.000 |
| Chronic heart failure | 23791 | 0.000 | 4272 | 0.000 | 17928 | 0.000 | 106 | 0.000 |
| Chronic liver disease | 23791 | 0.000 | 4272 | 0.000 | 17928 | 0.000 | 106 | 0.000 |
| COPD | 23791 | 0.000 | 4272 | 0.000 | 17928 | 0.000 | 106 | 0.000 |
| **Infection sources, n%** |  |  |  |  |  |  |  |  |
| Lung | 23791 | 0.000 | 4272 | 0.000 | 17928 | 0.000 | 106 | 0.000 |
| Intestinal | 23791 | 0.000 | 4272 | 0.000 | 17928 | 0.000 | 106 | 0.000 |
| Catheter related | 23791 | 0.000 | 4272 | 0.000 | 17928 | 0.000 | 106 | 0.000 |
| Urinary system | 23791 | 0.000 | 4272 | 0.000 | 17928 | 0.000 | 106 | 0.000 |
| Skin and soft tissue | 23791 | 0.000 | 4272 | 0.000 | 17928 | 0.000 | 106 | 0.000 |
| **Severity scale** |  |  |  |  |  |  |  |  |
| SOFA | 23791 | 0.000 | 4272 | 0.000 | 17928 | 0.000 | 106 | 0.000 |
| GCS | 18597 | 0.218 | 4268 | 0.001 | 17923 | 0.000 | 45 | 0.575 |
| **Vital signs** |  |  |  |  |  |  |  |  |
| HR_max, beats/min | 22249 | 0.065 | 4272 | 0.000 | 17928 | 0.000 | 102 | 0.038 |
| MAP_min, mmHg | 22177 | 0.068 | 4272 | 0.000 | 17928 | 0.000 | 106 | 0.000 |
| RR_max, times/min | 21979 | 0.076 | 4265 | 0.002 | 17919 | 0.001 | 106 | 0.000 |
| Temperature_max, °C | 22975 | 0.034 | 4269 | 0.001 | 17044 | 0.049 | 106 | 0.000 |
| **Interventions,n%** |  |  |  |  |  |  |  |  |
| KRT | 23791 | 0.000 | 4272 | 0.000 | 17928 | 0.000 | 106 | 0.000 |
| MV | 23791 | 0.000 | 4272 | 0.000 | 17928 | 0.000 | 106 | 0.000 |
| Norepinephrine | 23791 | 0.000 | 4272 | 0.000 | 17928 | 0.000 | 106 | 0.000 |
| Diuretic | 23791 | 0.000 | 4272 | 0.000 | 17928 | 0.000 | 106 | 0.000 |
| Furosemide dose, mg | 50 | 0.998 | 4272 | 0.000 | 17928 | 0.000 | 106 | 0.000 |
| Statin | 23791 | 0.000 | 4272 | 0.000 | 17928 | 0.000 | 106 | 0.000 |
| ACEI/ARBs | 23791 | 0.000 | 4272 | 0.000 | 17928 | 0.000 | 106 | 0.000 |
| Aminoglycoside | 23791 | 0.000 | 4272 | 0.000 | 17928 | 0.000 | 106 | 0.000 |
| Glycopeptide | 23791 | 0.000 | 4272 | 0.000 | 17928 | 0.000 | 106 | 0.000 |
| NSAIDs | 23791 | 0.000 | 4272 | 0.000 | 17928 | 0.000 | 106 | 0.000 |
| Acyclovir | 23791 | 0.000 | 4272 | 0.000 | 17928 | 0.000 | 106 | 0.000 |
| Fluid balance | 20637 | 0.133 | 4272 | 0.000 | 17553 | 0.021 | 102 | 0.038 |
| Urine output | 18150 | 0.237 | 4172 | 0.023 | 17803 | 0.007 | 106 | 0.000 |
| **Laboratory findings** |  |  |  |  |  |  |  |  |
| PaO2_min, mmHg | 13639 | 0.427 | 3338 | 0.219 | 14216 | 0.207 | 105 | 0.009 |
| ΔPaO2, mmHg | 6234 | 0.738 | 2388 | 0.441 | 8325 | 0.536 | 106 | 0.000 |
| Paco2_max, mmHg | 13473 | 0.434 | 3337 | 0.219 | 14216 | 0.207 | 105 | 0.009 |
| ΔPaco2, mmHg | 6124 | 0.743 | 2388 | 0.441 | 8323 | 0.536 | 106 | 0.000 |
| PH_min | 13382 | 0.438 | 3407 | 0.202 | 14216 | 0.207 | 105 | 0.009 |
| Lactate_max, mmol/L | 15333 | 0.356 | 3044 | 0.287 | 12927 | 0.279 | 105 | 0.009 |
| Lactate cleanrate | 5884 | 0.753 | 1477 | 0.654 | 5821 | 0.675 | 106 | 0.000 |
| BE_max, mmol/L | 10526 | 0.558 | 3337 | 0.219 | 14216 | 0.207 | 105 | 0.009 |
| Glucose_max, mg/dL | 23627 | 0.007 | 4244 | 0.007 | 17859 | 0.004 | 105 | 0.009 |
| BUN_max, mmol/L | 23462 | 0.014 | 4269 | 0.001 | 17910 | 0.001 | 104 | 0.019 |
| ΔSCr,mg/dL | 20985 | 0.118 | 3945 | 0.077 | 16022 | 0.106 | 106 | 0.000 |
| WBC_max, k/uL | 23268 | 0.022 | 4264 | 0.002 | 17902 | 0.001 | 95 | 0.104 |
| Platelets_max, k/uL | 23194 | 0.025 | 4264 | 0.002 | 17901 | 0.002 | 104 | 0.019 |
| RBC_min, k/uL | 23196 | 0.025 | 4257 | 0.004 | 17903 | 0.001 | 103 | 0.028 |
| MCHC_min, g/dL | 22813 | 0.041 | 4257 | 0.004 | 17903 | 0.001 | 103 | 0.028 |
| MCV_min, fL | 22820 | 0.041 | 4257 | 0.004 | 17903 | 0.001 | 84 | 0.208 |
| RDW_max, % | 21888 | 0.080 | 4257 | 0.004 | 17897 | 0.002 | 103 | 0.028 |
| Calcium_max, mEq/L | 23000 | 0.033 | 3998 | 0.064 | 15955 | 0.110 | 102 | 0.038 |
| Potassium_max, mEq/L | 23477 | 0.013 | 4216 | 0.013 | 17886 | 0.002 | 102 | 0.038 |
| Chloride_max, mEq/L | 23441 | 0.015 | 4266 | 0.001 | 17904 | 0.001 | 103 | 0.028 |
| Aniongap_max, mmol/L | 18812 | 0.209 | 4208 | 0.015 | 17861 | 0.004 | 104 | 0.019 |
| PT_max, seconds | 13220 | 0.444 | 3997 | 0.064 | 16851 | 0.060 | 103 | 0.028 |
| PTT_max, seconds | 9560 | 0.598 | 3990 | 0.066 | 16787 | 0.064 | 103 | 0.028 |

Max and min represented the maximum and minimum values during the first 24 hours after ICU admission, respectively. AKI: acute kidney injury; HR: heart rate; MAP: mean arterial pressure; Temp: temperature; COPD: chronic obstructive pulmonary disease; SOFA: Sequential Organ Failure Assessment; GCS: Glasgow Coma Scale; KRT: kidney replacement therapy; MV: mechanical ventilation; ACEI/ARBS: angiotensin-converting enzyme inhibitor/angiotensin receptor blocker; NSAIDS: Nonsteroidal Anti-inflammatory Drugs; PH: potential of hydrogen;PaCO2: partial pressure of carbon dioxide; PaO2: partial pressure of oxygen; BE: base excess; WBC: white blood cell;APTT: activated partial thromboplastin time; BUN: blood urea nitrogen; MCHC:mean corpuscular hemoglobin concentration; MCV: mean corpuscular volume; RDW: Red blood cell distribution width; PT: Prothrombin time;
