# Supplementary material for: Explainable Machine Learning Model for Predicting Persistent Sepsis-Associated Acute Kidney Injury: Development and Validation Study
Source: J Med Internet Res. 2025 Apr 28;27:e62932. doi: 10.2196/62932 (PMC12070005; doi:10.2196/62932)
Supplement: Multimedia Appendix 2 [file jmir_v27i1e62932_app2.docx]

**
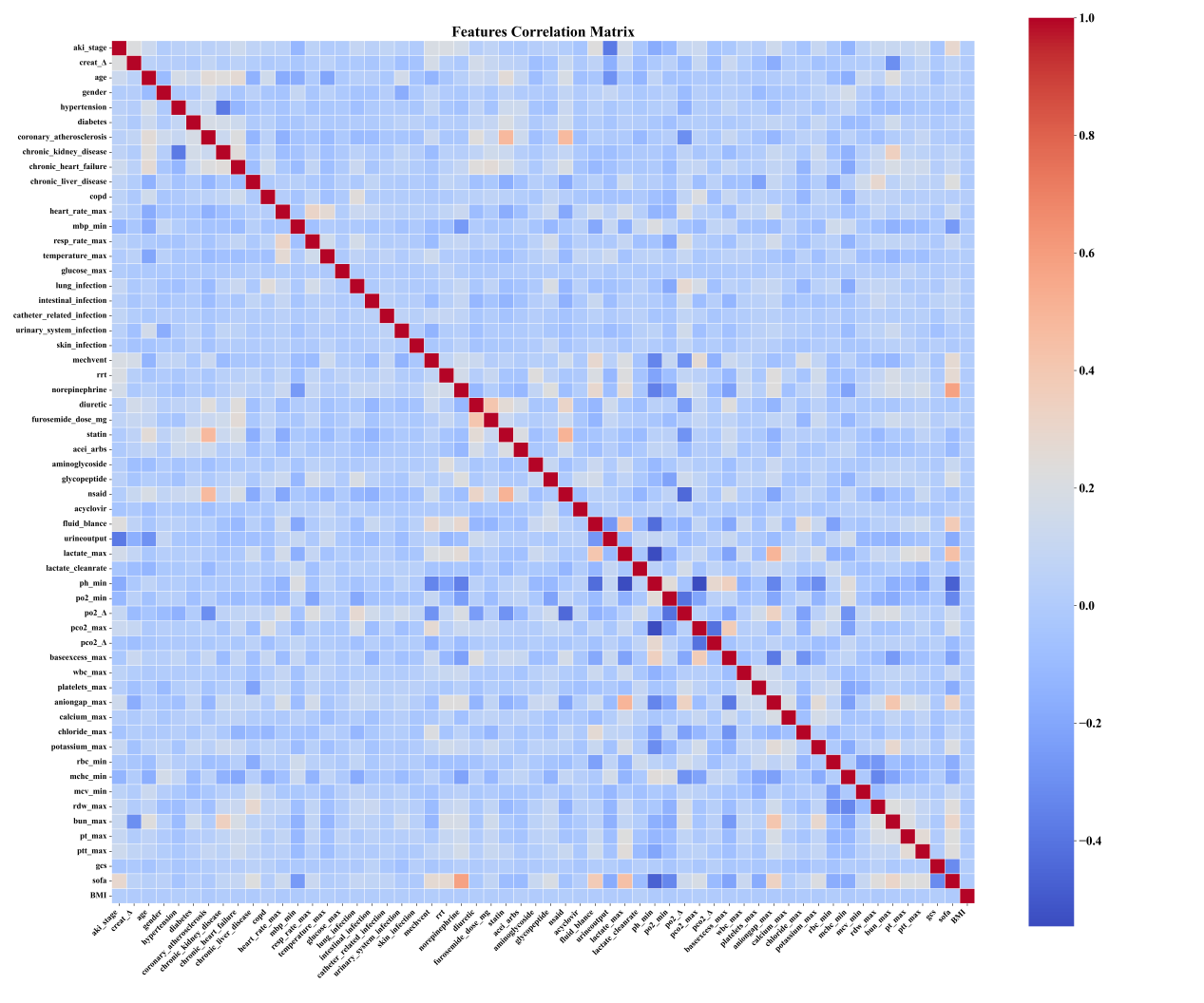
Multimedia Appendix 2. Heat map of Spearman correlation analyses among variables.** Variables of critically ill children from the derivation cohort were included in Spearman correlation analyses. Variables in Multimedia Appendix 1 (except variables with a missing percentage exceeding 25%) were chosen for the Spearman correlation analyses, and the correlation coefficient values were displayed as a heat map. SA-AKI: sepsis associated acute kidney injury; HR: heart rate; MAP: mean arterial pressure; Temp: temperature; COPD: chronic obstructive pulmonary disease; SOFA: Sequential Organ Failure Assessment; GCS: Glasgow Coma Scale; KRT: kidney replacement therapy; MV: mechanical ventilation; ACEI/ARBS: angiotensin-converting enzyme inhibitor/angiotensin receptor blocker; NSAIDS: Nonsteroidal Anti-inflammatory Drugs; PH: potential of hydrogen;PaCO2: partial pressure of carbon dioxide; PaO2: partial pressure of oxygen; BE: base excess; WBC: white blood cell; APTT: activated partial thromboplastin time; BUN: blood urea nitrogen; MCHC:mean corpuscular hemoglobin concentration; MCV: mean corpuscular volume; RDW: Red blood cell distribution width; PT: Prothrombin time;
