# Supplementary material for: Explainable Machine Learning Model for Predicting Persistent Sepsis-Associated Acute Kidney Injury: Development and Validation Study
Source: J Med Internet Res. 2025 Apr 28;27:e62932. doi: 10.2196/62932 (PMC12070005; doi:10.2196/62932)
Supplement: Multimedia Appendix 3 [file jmir_v27i1e62932_app3.docx]

**Multimedia Appendix 3.** Comparison of demographic and clinical characteristics among the training, internal validation, and external validation cohorts.

| Variables | **Internal validation(n=3586)** | **Training(n=14342)** | **Derivation (n=17928)** | **External validation 1(n=23791)** | **External validation 2(n=4272)** | **External validation 3(n=106)** |
| --- | --- | --- | --- | --- | --- | --- |
| AKI n (%) | 1605 [44.76%] | 6476 [45.15%] | 8081 [45.07%] | 6091 [25.60%] | 1848 [43.26%] | 36 [33.96%] |
| AKI stage | 67.84 [56.31-79.05] | 67.99 [56.74-79.25] | 67.96 [56.63-79.19] | 67.00 [55.00-79.00] | 69.45 [54.50-80.43] | 69.50 [59.50-78.00] |
| AKI stage 1 | 472 [13.16%] | 1827 [12.74%] | 2299 [12.82%] | 2609 [10.97%] | 439 [10.28%] | 8 [7.55%] |
| AKI stage 2 | 894 [24.93%] | 3651 [25.46%] | 4545 [25.35%] | 1879 [7.90%] | 1044 [24.44%] | 15 [14.15%] |
| AKI stage 3 | 239 [6.66%] | 998 [6.96%] | 1237 [6.90%] | 1603 [6.74%] | 365 [8.54%] | 13 [12.26%] |
| **Demographic data** |  |  |  |  |  |  |
| BMI, kg/m2 | 27.49 [23.76-32.01] | 27.55 [23.94-32.08] | 27.54 [23.89-32.06] | 27.04 [22.89-32.75] | 26.48 [23.44-30.51] | 22.68 [20.76-24.73] |
| Gender, Male, n% | 2067 [57.64%] | 8332 [58.10%] | 10399 [58.00%] | 12359.591081872719 [51.95%] | 2419 [56.62%] | 68 [64.15%] |
| **Commodities，n%** |  |  |  |  |  |  |
| Hypertension | 1648 [45.96%] | 6588 [45.94%] | 8236 [45.94%] | 13362 [56.16%] | 1607 [37.62%] | 61 [57.55%] |
| Diabetes | 957 [26.69%] | 4076 [28.42%] | 5033 [28.07%] | 7952 [33.42%] | 1034 [24.20%] | 28 [26.42%] |
| Coronary atherosclerosis | 1071 [29.87%] | 4391 [30.62%] | 5462 [30.47%] | 1628 [6.84%] | 761 [17.81%] | 6 [5.66%] |
| Chronic kidney disease | 523 [14.58%] | 2182 [15.21%] | 2705 [15.09%] | 2019 [8.49%] | 226 [5.29%] | 6 [5.66%] |
| Chronic heart failure | 896 [24.99%] | 3768 [26.27%] | 4664 [26.02%] | 3056 [12.85%] | 1572 [36.80%] | 7 [6.60%] |
| Chronic liver disease | 331 [9.23%] | 1335 [9.31%] | 1666 [9.29%] | 728 [3.06%] | 286 [6.69%] | 7 [6.60%] |
| COPD | 546 [15.23%] | 2159 [15.05%] | 2705 [15.09%] | 5731 [24.09%] | 683 [15.99%] | 5 [4.72%] |
| **Infection sources, n%** |  |  |  |  |  |  |
| Lung | 1393 [38.85%] | 5669 [39.53%] | 7062 [39.39%] | 11606 [48.78%] | 2008 [47.00%] | 35 [33.02%] |
| Intestinal | 462 [12.88%] | 1900 [13.25%] | 2362 [13.17%] | 1743 [7.33%] | 605 [14.16%] | 3 [2.83%] |
| Catheter related | 257 [7.17%] | 906 [6.32%] | 1163 [6.49%] | 224 [0.94%] | 207 [4.85%] | 52 [49.06%] |
| Urinary system | 645 [17.99%] | 2676 [18.66%] | 3321 [18.52%] | 5475 [23.01%] | 840 [19.66%] | 11 [10.38%] |
| Skin and soft tissue | 166 [4.63%] | 677 [4.72%] | 843 [4.70%] | 1984 [8.34%] | 268 [6.27%] | 2 [1.89%] |
| **Severity scale** |  |  |  |  |  |  |
| SOFA | 5.00 [3.00-7.00] | 5.00 [3.00-7.00] | 5.00 [3.00-7.00] | 6.00 [4.00-8.00] | 5.00 [3.00-7.00] | 10.00 [7.00-12.00] |
| GCS | 15.00 [13.00-15.00] | 15.00 [13.00-15.00] | 15.00 [13.00-15.00] | 14.00 [10.00-15.00] | 15.00 [14.00-15.00] | 10.74 [9.56-12.13] |
| **Vital signs** |  |  |  |  |  |  |
| HR_max, beats/min | 103.00 [90.00-117.00] | 103.00 [91.00-118.00] | 103.00 [91.00-118.00] | 110.00 [96.00-125.00] | 107.00 [93.00-122.00] | 121.35 [108.00-135.75] |
| MAP_min, mmHg | 57.00 [51.00-63.00] | 58.00 [51.00-64.00] | 58.00 [51.00-64.00] | 59.00 [51.00-67.00] | 57.33 [50.00-64.00] | 64.00 [57.25-68.75] |
| RR_max, times/min | 27.00 [24.00-32.00] | 27.00 [24.00-32.00] | 27.00 [24.00-32.00] | 29.00 [24.00-34.00] | 28.00 [24.00-32.00] | 23.00 [20.00-27.00] |
| Temp_max, °C | 37.33 [37.00-37.90] | 37.39 [37.00-37.90] | 37.38 [37.00-37.90] | 37.40 [37.00-38.10] | 37.67 [37.11-38.28] | 37.80 [37.02-38.40] |
| **Interventions** |  |  |  |  |  |  |
| KRT, n% | 112 [3.12%] | 458 [3.19%] | 570 [3.18%] | 366 [1.54%] | 100 [2.34%] | 35 [33.02%] |
| MV, n% | 1944 [54.21%] | 7811 [54.46%] | 9755 [54.41%] | 10130 [42.58%] | 2538 [59.41%] | 82 [77.36%] |
| Norepinephrine% | 847 [23.62%] | 3365 [23.46%] | 4212 [23.49%] | 6200 [26.06%] | 793 [18.56%] | 98 [92.45%] |
| Diuretic, n% | 1101 [30.70%] | 4508 [31.43%] | 5609 [31.29%] | 3368 [14.16%] | 1370 [32.07%] | 20 [18.87%] |
|  |  |  |  |  |  |  |
| Statin, n% | 1268 [35.36%] | 5156 [35.95%] | 6424 [35.83%] | 2168 [9.11%] | 953 [22.31%] | 0 [0.00%] |
| ACEI/ARBs, n% | 296 [8.25%] | 1328 [9.26%] | 1624 [9.06%] | 1034 [4.35%] | 474 [11.10%] | 0 [0.00%] |
| Aminoglycoside, n% | 109 [3.04%] | 418 [2.91%] | 527 [2.94%] | 84 [0.35%] | 207 [4.85%] | 1 [0.94%] |
| Glycopeptide, n% | 1980 [55.21%] | 8047 [56.11%] | 10027 [55.93%] | 8705 [36.59%] | 2161 [50.59%] | 0 [0.00%] |
| NSAIDS, n% | 1466 [40.88%] | 5887 [41.05%] | 7353 [41.01%] | 3934 [16.54%] | 1496 [35.02%] | 0 [0.00%] |
| Acyclovir, n% | 128 [3.57%] | 593 [4.13%] | 721 [4.02%] | 109 [0.46%] | 131 [3.07%] | 2 [1.89%] |
| Furosemide dose, mg | 0.00 [0.00-0.00] | 0.00 [0.00-0.00] | 0.00 [0.00-0.00] | 1266.30 [1261.40-1271.09] | 0.00 [0.00-0.00] | 0.00 [0.00-0.00] |
| Fluid balance | 2858.00 [788.50-5497.50] | 2824.50 [686.75-5395.00] | 2830.00 [706.00-5422.25] | 0.00 [-1026.70-1498.25] | -1089.00 [-2015.00--38.00] | 2104.50 [888.50-3148.75] |
| **Laboratory findings** |  |  |  |  |  |  |
| PaO_2__min, mmHg | 80.00 [51.43-106.00] | 80.47 [51.00-105.00] | 80.19 [51.00-105.00] | 88.00 [69.40-103.93] | 88.00 [63.00-110.18] | 100.10 [77.55-131.60] |
| ΔPaO_2_, mmHg | -0.65 [-3.00-1.80] | -0.77 [-3.14-1.56] | -0.78 [-3.07-1.42] | -0.85 [-5.83-3.85] | -0.23 [-3.72-2.00] | 1.80 [-5.10-10.15] |
| PaCO2_max, mmHg | 45.70 [40.75-52.00] | 46.00 [40.97-52.00] | 45.93 [41.00-51.68] | 41.06 [34.28-50.00] | 44.37 [39.00-52.00] | 40.55 [36.52-46.15] |
| ΔPaO2, mmHg | -47.64 [-132.00-0.00] | -48.31 [-133.00--2.70] | -50.43 [-135.00--3.00] | -15.00 [-38.30-5.95] | -35.65 [-80.84-0.00] | -25.00 [-151.78-22.15] |
| PH_min | 7.33 [7.27-7.38] | 7.33 [7.27-7.38] | 7.33 [7.27-7.38] | 7.36 [7.29-7.41] | 7.34 [7.26-7.39] | 7.33 [7.28-7.37] |
| Lactate_max, mmol/L | 2.20 [1.50-3.30] | 2.20 [1.50-3.20] | 2.20 [1.50-3.20] | 2.10 [1.30-3.33] | 2.40 [1.60-3.85] | 2.30 [1.52-3.98] |
| Lactate cleanrate | 0.10 [-0.00-0.22] | 0.11 [0.00-0.22] | 0.09 [-0.00-0.20] | 0.33 [0.11-0.52] | 0.20 [0.08-0.30] | -0.00 [-0.04-0.02] |
| BE_max, mmol/L | 0.25 [-1.00-3.00] | 0.00 [-1.03-3.00] | 0.02 [-1.00-2.51] | -0.94 [-4.04-2.00] | 0.10 [-1.31-3.00] | -2.10 [-5.38-1.55] |
| Glucose_max, mg/dL | 166.00 [131.00-212.00] | 166.00 [132.00-211.00] | 166.00 [132.00-211.00] | 163.00 [127.00-227.00] | 165.00 [132.00-214.00] | 9.15 [7.45-11.73] |
| BUN_max, mmol/L | 20.00 [15.00-31.00] | 20.00 [15.00-31.75] | 20.00 [15.00-31.00] | 25.00 [16.00-41.00] | 23.00 [16.00-39.00] | 34.95 [29.45-40.38] |
| ΔScr,mg/dL | 0.00 [-0.20-0.10] | 0.00 [-0.20-0.10] | 0.00 [-0.20-0.10] | -0.10 [-0.30-0.10] | -0.04 [-0.20-0.10] | -0.03 [-0.23-0.10] |
| WBC_max, k/uL | 13.70 [9.80-18.70] | 13.70 [9.80-18.40] | 13.70 [9.80-18.50] | 13.80 [9.50-19.30] | 13.70 [9.70-18.82] | 21.60 [19.33-23.40] |
| Platelets_max, k/uL | 199.00 [145.00-272.00] | 199.00 [145.00-268.00] | 199.00 [145.00-269.00] | 214.28 [153.00-289.00] | 227.00 [159.00-306.00] | 144.15 [141.75-148.03] |
| RBC_min, k/uL | 3.28 [2.82-3.82] | 3.29 [2.80-3.80] | 3.28 [2.81-3.80] | 3.56 [3.06-4.06] | 3.38 [2.96-3.82] | 3.48 [3.06-4.02] |
| MCHC_min, g/dL | 32.70 [31.60-33.70] | 32.70 [31.60-33.70] | 32.70 [31.60-33.70] | 32.53 [31.60-33.40] | 33.40 [32.30-34.40] | 325.00 [315.25-335.00] |
| MCV_min, fL | 90.00 [86.00-94.00] | 90.00 [86.00-94.00] | 90.00 [86.00-94.00] | 90.00 [86.00-94.10] | 89.00 [85.00-93.00] | 93.78 [90.93-95.88] |
| RDW_max, % | 14.60 [13.60-16.10] | 14.60 [13.60-16.20] | 14.60 [13.60-16.20] | 15.30 [14.10-16.90] | 14.90 [13.80-16.60] | 14.50 [13.53-16.27] |
| Calcium_max, mEq/L | 8.40 [8.00-8.90] | 8.40 [8.00-8.90] | 8.40 [8.00-8.90] | 8.60 [8.00-9.10] | 8.50 [8.00-9.00] | 9.59 [5.18-13.92] |
| Potassium_max, mEq/L | 4.40 [4.10-4.90] | 4.40 [4.10-4.90] | 4.40 [4.10-4.90] | 4.30 [3.90-4.80] | 4.40 [4.00-4.90] | 150.00 [84.00-214.75] |
| Chloride_max, mEq/L | 107.00 [103.00-111.00] | 107.00 [104.00-111.00] | 107.00 [104.00-111.00] | 106.00 [101.48-110.00] | 107.00 [103.00-111.00] | 0.43 [0.22-0.71] |
| Aniongap_max, mmol/L | 15.00 [13.00-18.00] | 15.00 [13.00-18.00] | 15.00 [13.00-18.00] | 12.00 [9.09-15.61] | 16.00 [13.00-19.00] | 3.52 [3.22-3.80] |
| PT_max, seconds | 14.90 [13.10-17.60] | 14.90 [13.10-17.52] | 14.90 [13.10-17.60] | 16.70 [14.07-20.53] | 14.70 [13.50-17.10] | 117.15 [78.58-195.27] |
| PTT_max, seconds | 33.10 [28.52-41.89] | 32.80 [28.50-41.50] | 32.90 [28.50-41.60] | 37.01 [31.46-43.04] | 33.30 [27.80-45.20] | 12.93 [8.89-19.51] |
|  |  |  |  |  |  |  |

Continuous values were presented as median [interquartile range]. Categorical values were presented as number (percentage). AKI: acute kidney injury; HR: heart rate; MAP: mean arterial pressure; Temp: temperature; COPD: chronic obstructive pulmonary disease; SOFA: Sequential Organ Failure Assessment; GCS: Glasgow Coma Scale; KRT: kidney replacement therapy; MV: mechanical ventilation; ACEI/ARBS: angiotensin-converting enzyme inhibitor/angiotensin receptor blocker; NSAIDS: Nonsteroidal Anti-inflammatory Drugs; PH: potential of hydrogen;PaCO2: partial pressure of carbon dioxide; PaO2: partial pressure of oxygen; BE: base excess; WBC: white blood cell; APTT: activated partial thromboplastin time; BUN: blood urea nitrogen; MCHC:mean corpuscular hemoglobin concentration; MCV: mean corpuscular volume; RDW: Red blood cell distribution width; PT: Prothrombin time;
