# Supplementary material for: Explainable Machine Learning Model for Predicting Persistent Sepsis-Associated Acute Kidney Injury: Development and Validation Study
Source: J Med Internet Res. 2025 Apr 28;27:e62932. doi: 10.2196/62932 (PMC12070005; doi:10.2196/62932)
Supplement: Multimedia Appendix 4 [file jmir_v27i1e62932_app4.docx]

**Multimedia Appendix 4.** Performance of the machine learning models for persistent sepsis-associated acute kidney injury prediction.

| Models | AUROC | AUPRC | Accuracy | F1 Score | Precision | Sensitivity | Specificity | NPV |
| --- | --- | --- | --- | --- | --- | --- | --- | --- |
| GBM | 0.872 | 0.826 | 0.772 | 0.767 | 0.706 | 0.840 | 0.717 | 0.847 |
| CatBoost | 0.870 | 0.819 | 0.774 | 0.767 | 0.711 | 0.832 | 0.726 | 0.842 |
| XGBoost | 0.859 | 0.805 | 0.768 | 0.755 | 0.716 | 0.798 | 0.743 | 0.820 |
| Logistic Regression | 0.859 | 0.810 | 0.759 | 0.739 | 0.717 | 0.763 | 0.756 | 0.798 |
| SVM | 0.858 | 0.808 | 0.764 | 0.755 | 0.705 | 0.814 | 0.724 | 0.827 |
| ANN | 0.834 | 0.761 | 0.733 | 0.701 | 0.702 | 0.700 | 0.760 | 0.757 |
| KNN | 0.751 | 0.663 | 0.688 | 0.631 | 0.670 | 0.597 | 0.762 | 0.700 |
| DT | 0.713 | 0.610 | 0.718 | 0.680 | 0.689 | 0.672 | 0.755 | 0.739 |

The indexes represented the performance of ML models in the internal validation cohort.SA-AKI: sepsis associated acute kidney injury; AUC: area under the receiver-operating-characteristic curve; NPV: negative predictive value; GBM: gradient boosting machine; CatBoost: categorical boosting; XGboost: extreme gradient boost; SVM: support vector machine; ANN: artificial neutral network; DT: decision tree; KNN: K-nearest neighbor; ML: machine learning.
