# Supplementary material for: Explainable Machine Learning Model for Predicting Persistent Sepsis-Associated Acute Kidney Injury: Development and Validation Study
Source: J Med Internet Res. 2025 Apr 28;27:e62932. doi: 10.2196/62932 (PMC12070005; doi:10.2196/62932)
Supplement: Multimedia Appendix 5 [file jmir_v27i1e62932_app5.docx]

C

B

A


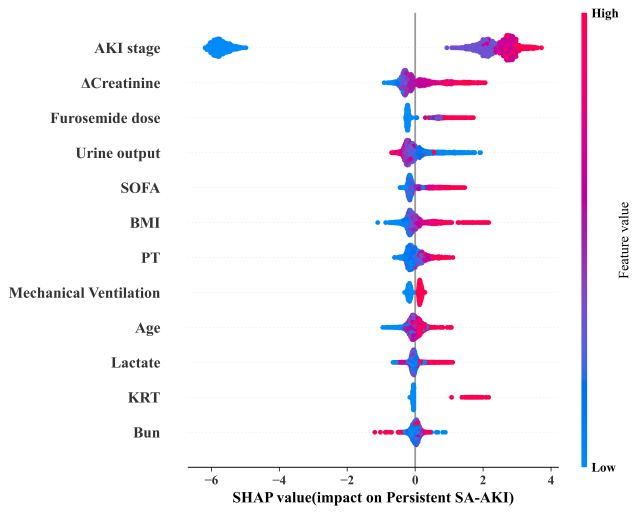

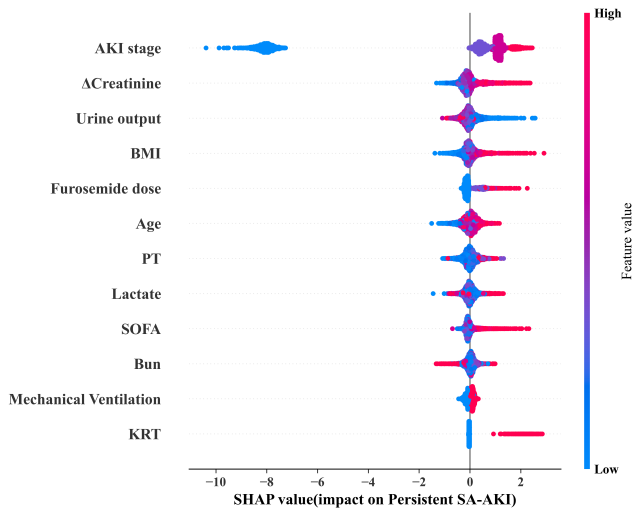

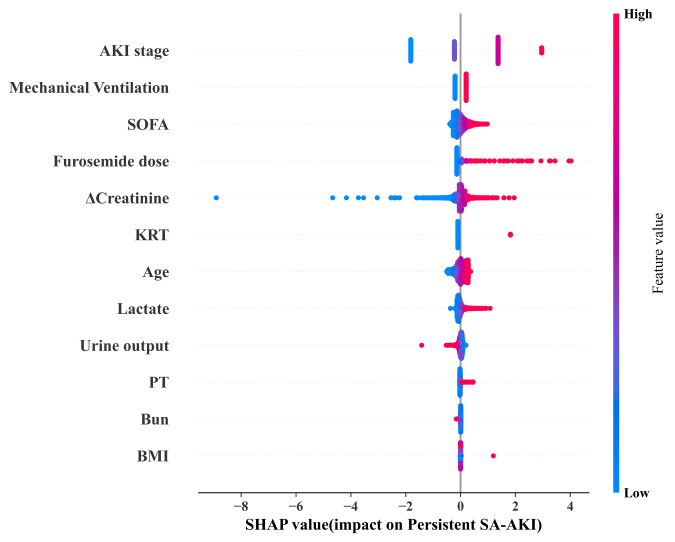


D


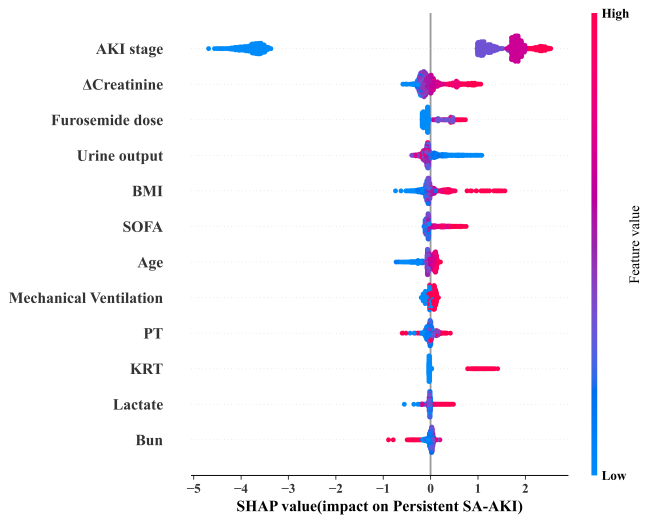


**Multimedia Appendix 5. Shapley Additive Explanations (SHAP) summary dot plots of the 12 features of the top 4 best performing machine learning models.** (A)CatBoost, (B)XGBoost, (C) Logistic Regression, (D)GBM. These plots represented the feature important rank of ML models in the internal validation cohort. The probability of persistent SA-AKI development increases with the SHAP value of a feature. A dot is made for each feature attribution value in the model for each single patient, so each patient has one dot on every line for each feature. The actual values of the features for each patient are shown by the colors of the dots: red means a higher actual value, and blue means a lower actual value. The dots are stacked vertically to show density. Max and min represented the maximum and minimum values during the first 24 hours after ICU admission, respectively. SA-AKI: sepsis associated acute kidney injury; AKI: acute kidney injury; △Creatinine: Changes in creatinine within 24 hours; BMI: body mass Index; SOFA: sequential organ failure assessment; KRT: kidney renal therapy; PT:PT:Prothrombin time; GBM: gradient boosting machine; CatBoost: categorical boosting; XGboost: extreme gradient boost.
