# Supplementary material for: Explainable Machine Learning Model for Predicting Persistent Sepsis-Associated Acute Kidney Injury: Development and Validation Study
Source: J Med Internet Res. 2025 Apr 28;27:e62932. doi: 10.2196/62932 (PMC12070005; doi:10.2196/62932)
Supplement: Multimedia Appendix 6 [file jmir_v27i1e62932_app6.docx]

**Multimedia Appendix 6.** Performance of the gradient boosting machine model with varied numbers of features for persistent sepsis-associated acute kidney injury prediction.

| **Number** | **Removed Feature** | **AUROC** | **P-Value** | **95% CI** | **AUPRC** | **Accuracy** | **F1 Score** | **Precision** | **Recall** | **Specificity** | **NPV** |
| --- | --- | --- | --- | --- | --- | --- | --- | --- | --- | --- | --- |
| 58 | acei_arbs |  |  |  |  |  |  |  |  |  |  |
| 57 | chronic_kidney_disease | 0.872 | 0.761 | 0.8613, 0.8723 | 0.826 | 0.772 | 0.767 | 0.706 | 0.840 | 0.717 | 0.847 |
| 56 | copd | 0.872 | 0.319 | 0.8613, 0.8723 | 0.826 | 0.772 | 0.767 | 0.706 | 0.840 | 0.717 | 0.847 |
| 55 | diabetes | 0.872 | 0.319 | 0.8613, 0.8723 | 0.826 | 0.772 | 0.767 | 0.706 | 0.840 | 0.717 | 0.847 |
| 54 | glycopeptide | 0.872 | 0.806 | 0.8612, 0.8723 | 0.826 | 0.772 | 0.767 | 0.706 | 0.840 | 0.717 | 0.847 |
| 53 | hypertension | 0.872 | 0.761 | 0.8613, 0.8723 | 0.826 | 0.772 | 0.767 | 0.706 | 0.840 | 0.717 | 0.847 |
| 52 | intestinal_infection | 0.872 | 0.761 | 0.8613, 0.8723 | 0.826 | 0.772 | 0.767 | 0.706 | 0.840 | 0.717 | 0.847 |
| 51 | urinary_system_infection | 0.872 | 0.761 | 0.8613, 0.8723 | 0.826 | 0.772 | 0.767 | 0.706 | 0.840 | 0.717 | 0.847 |
| 50 | gender | 0.872 | 0.761 | 0.8613, 0.8723 | 0.826 | 0.772 | 0.767 | 0.706 | 0.840 | 0.717 | 0.847 |
| 49 | norepinephrine | 0.872 | 0.761 | 0.8613, 0.8723 | 0.826 | 0.772 | 0.767 | 0.706 | 0.840 | 0.717 | 0.847 |
| 48 | aminoglycoside | 0.872 | 0.319 | 0.8613, 0.8723 | 0.826 | 0.772 | 0.767 | 0.706 | 0.840 | 0.717 | 0.847 |
| 47 | acyclovir | 0.872 | 0.328 | 0.8613, 0.8723 | 0.826 | 0.772 | 0.767 | 0.706 | 0.840 | 0.717 | 0.847 |
| 46 | potassium_max | 0.872 | 0.575 | 0.8614, 0.8724 | 0.827 | 0.771 | 0.767 | 0.705 | 0.840 | 0.715 | 0.846 |
| 45 | platelets_max | 0.872 | 0.996 | 0.8613, 0.8723 | 0.826 | 0.771 | 0.766 | 0.705 | 0.838 | 0.716 | 0.845 |
| 44 | gcs | 0.873 | 0.247 | 0.8618, 0.8728 | 0.828 | 0.774 | 0.769 | 0.708 | 0.841 | 0.719 | 0.848 |
| 43 | skin_infection | 0.873 | 0.523 | 0.8617, 0.8726 | 0.828 | 0.778 | 0.773 | 0.712 | 0.847 | 0.722 | 0.853 |
| 42 | statin | 0.873 | 0.409 | 0.8618, 0.8728 | 0.828 | 0.777 | 0.772 | 0.710 | 0.846 | 0.720 | 0.852 |
| 41 | chronic_liver_disease | 0.873 | 0.409 | 0.8618, 0.8728 | 0.828 | 0.777 | 0.772 | 0.710 | 0.846 | 0.720 | 0.852 |
| 40 | chloride_max | 0.873 | 0.552 | 0.8616, 0.8726 | 0.827 | 0.775 | 0.771 | 0.708 | 0.847 | 0.716 | 0.853 |
| 39 | calcium_max | 0.872 | 0.803 | 0.8611, 0.8721 | 0.827 | 0.772 | 0.768 | 0.705 | 0.843 | 0.715 | 0.849 |
| 38 | wbc_max | 0.872 | 0.830 | 0.8611, 0.8722 | 0.827 | 0.772 | 0.768 | 0.705 | 0.842 | 0.715 | 0.848 |
| 37 | aniongap_max | 0.872 | 0.647 | 0.8610, 0.8720 | 0.827 | 0.774 | 0.769 | 0.708 | 0.842 | 0.719 | 0.849 |
| 36 | heart_rate_max | 0.872 | 0.507 | 0.8609, 0.8719 | 0.827 | 0.777 | 0.773 | 0.710 | 0.847 | 0.719 | 0.853 |
| 35 | rbc_min | 0.873 | 0.138 | 0.8621, 0.8731 | 0.828 | 0.773 | 0.768 | 0.707 | 0.840 | 0.718 | 0.847 |
| 34 | diuretic | 0.873 | 0.127 | 0.8622, 0.8732 | 0.828 | 0.772 | 0.767 | 0.707 | 0.838 | 0.718 | 0.846 |
| 33 | fluid_blance | 0.873 | 0.110 | 0.8624, 0.8733 | 0.829 | 0.774 | 0.769 | 0.707 | 0.844 | 0.717 | 0.850 |
| 32 | mchc_min | 0.873 | 0.376 | 0.8619, 0.8728 | 0.828 | 0.772 | 0.767 | 0.707 | 0.839 | 0.718 | 0.846 |
| 31 | pco2_Δ | 0.873 | 0.596 | 0.8617, 0.8726 | 0.828 | 0.775 | 0.771 | 0.708 | 0.845 | 0.718 | 0.851 |
| 30 | mbp_min | 0.873 | 0.721 | 0.8616, 0.8726 | 0.829 | 0.774 | 0.769 | 0.708 | 0.840 | 0.719 | 0.848 |
| 29 | resp_rate_max | 0.873 | 0.623 | 0.8617, 0.8727 | 0.828 | 0.775 | 0.771 | 0.709 | 0.845 | 0.718 | 0.852 |
| 28 | coronary_atherosclerosis | 0.873 | 0.580 | 0.8617, 0.8727 | 0.829 | 0.774 | 0.769 | 0.709 | 0.840 | 0.720 | 0.848 |
| 27 | glucose_max | 0.872 | 0.747 | 0.8610, 0.8720 | 0.827 | 0.771 | 0.766 | 0.705 | 0.839 | 0.716 | 0.846 |
| 26 | po2_min | 0.872 | 0.521 | 0.8607, 0.8717 | 0.828 | 0.769 | 0.764 | 0.703 | 0.837 | 0.714 | 0.844 |
| 25 | nsaid | 0.872 | 0.777 | 0.8610, 0.8720 | 0.827 | 0.772 | 0.767 | 0.706 | 0.840 | 0.717 | 0.847 |
| 24 | chronic_heart_failure | 0.871 | 0.337 | 0.8603, 0.8714 | 0.827 | 0.770 | 0.766 | 0.704 | 0.839 | 0.715 | 0.846 |
| 23 | ph_min | 0.871 | 0.082 | 0.8596, 0.8707 | 0.826 | 0.771 | 0.767 | 0.705 | 0.840 | 0.716 | 0.847 |
| 22 | lactate_cleanrate | 0.871 | 0.276 | 0.8602, 0.8712 | 0.826 | 0.771 | 0.766 | 0.705 | 0.839 | 0.716 | 0.846 |
| 21 | catheter_related_infection | 0.871 | 0.192 | 0.8599, 0.8710 | 0.826 | 0.773 | 0.768 | 0.706 | 0.842 | 0.716 | 0.849 |
| 20 | temperature_max | 0.871 | 0.347 | 0.8601, 0.8712 | 0.827 | 0.774 | 0.770 | 0.708 | 0.843 | 0.719 | 0.850 |
| 19 | mcv_min | 0.871 | 0.210 | 0.8597, 0.8708 | 0.826 | 0.774 | 0.769 | 0.708 | 0.842 | 0.719 | 0.849 |
| 18 | baseexcess_max | 0.871 | 0.314 | 0.8600, 0.8711 | 0.826 | 0.772 | 0.768 | 0.707 | 0.840 | 0.718 | 0.847 |
| 17 | pco2_max | 0.871 | 0.237 | 0.8597, 0.8708 | 0.826 | 0.776 | 0.771 | 0.710 | 0.844 | 0.721 | 0.851 |
| 16 | lung_infection | 0.870 | 0.048 | 0.8586, 0.8697 | 0.825 | 0.772 | 0.768 | 0.706 | 0.844 | 0.715 | 0.849 |
| 15 | rdw_max | 0.870 | 0.091 | 0.8589, 0.8700 | 0.825 | 0.774 | 0.770 | 0.707 | 0.844 | 0.717 | 0.850 |
| 14 | ptt_max | 0.870 | 0.102 | 0.8588, 0.8699 | 0.823 | 0.773 | 0.769 | 0.705 | 0.846 | 0.713 | 0.851 |
| 13 | po2_Δ | 0.869 | 0.025 | 0.8576, 0.8688 | 0.822 | 0.772 | 0.767 | 0.706 | 0.840 | 0.716 | 0.847 |
| 12 | lactate_max | 0.870 | 0.130 | 0.8587, 0.8698 | 0.824 | 0.769 | 0.764 | 0.704 | 0.836 | 0.716 | 0.843 |
| 11 | bun_max | 0.869 | 0.042 | 0.8583, 0.8695 | 0.824 | 0.771 | 0.766 | 0.706 | 0.837 | 0.718 | 0.845 |
| 10 | pt_max | 0.869 | 0.039 | 0.8575, 0.8687 | 0.823 | 0.772 | 0.768 | 0.706 | 0.842 | 0.716 | 0.848 |
| 9 | age | 0.868 | 0.028 | 0.8572, 0.8684 | 0.822 | 0.772 | 0.767 | 0.706 | 0.840 | 0.717 | 0.847 |
| 8 | mechvent | 0.866 | 0.001 | 0.8547, 0.8660 | 0.820 | 0.766 | 0.763 | 0.699 | 0.840 | 0.707 | 0.845 |
| 7 | rrt | 0.865 | 0.000 | 0.8538, 0.8652 | 0.818 | 0.767 | 0.765 | 0.698 | 0.845 | 0.703 | 0.849 |
| 6 | sofa | 0.864 | 0.000 | 0.8529, 0.8643 | 0.816 | 0.767 | 0.764 | 0.699 | 0.844 | 0.705 | 0.848 |
| 5 | BMI | 0.863 | 0.000 | 0.8513, 0.8628 | 0.814 | 0.766 | 0.764 | 0.696 | 0.845 | 0.701 | 0.849 |
| 4 | furosemide_dose_mg | 0.859 | 0.000 | 0.8479, 0.8595 | 0.812 | 0.766 | 0.767 | 0.692 | 0.860 | 0.689 | 0.859 |
| 3 | urineoutput | 0.855 | 0.000 | 0.8436, 0.8554 | 0.802 | 0.767 | 0.767 | 0.693 | 0.858 | 0.693 | 0.858 |
| 2 | creat_Δ | 0.854 | 0.000 | 0.8419, 0.8538 | 0.792 | 0.764 | 0.763 | 0.693 | 0.849 | 0.696 | 0.850 |
| 1 | aki_stage | 0.828 | 0.000 | 0.8159, 0.8282 | 0.717 | 0.751 | 0.783 | 0.643 | 1.000 | 0.550 | 1.000 |

The indexes represented the performance of the GBM model with varied numbers of features in the internal validation cohort. SA-AKI: sepsis associated acute kidney injury; AUC: area under the receiver-operating-characteristic curve; NPV: negative predictive value; PPV: positive predictive value; GBM: gradient boosting machine.
