# Supplementary material for: Explainable Machine Learning Model for Predicting Persistent Sepsis-Associated Acute Kidney Injury: Development and Validation Study
Source: J Med Internet Res. 2025 Apr 28;27:e62932. doi: 10.2196/62932 (PMC12070005; doi:10.2196/62932)
Supplement: Multimedia Appendix 7 [file jmir_v27i1e62932_app7.docx]

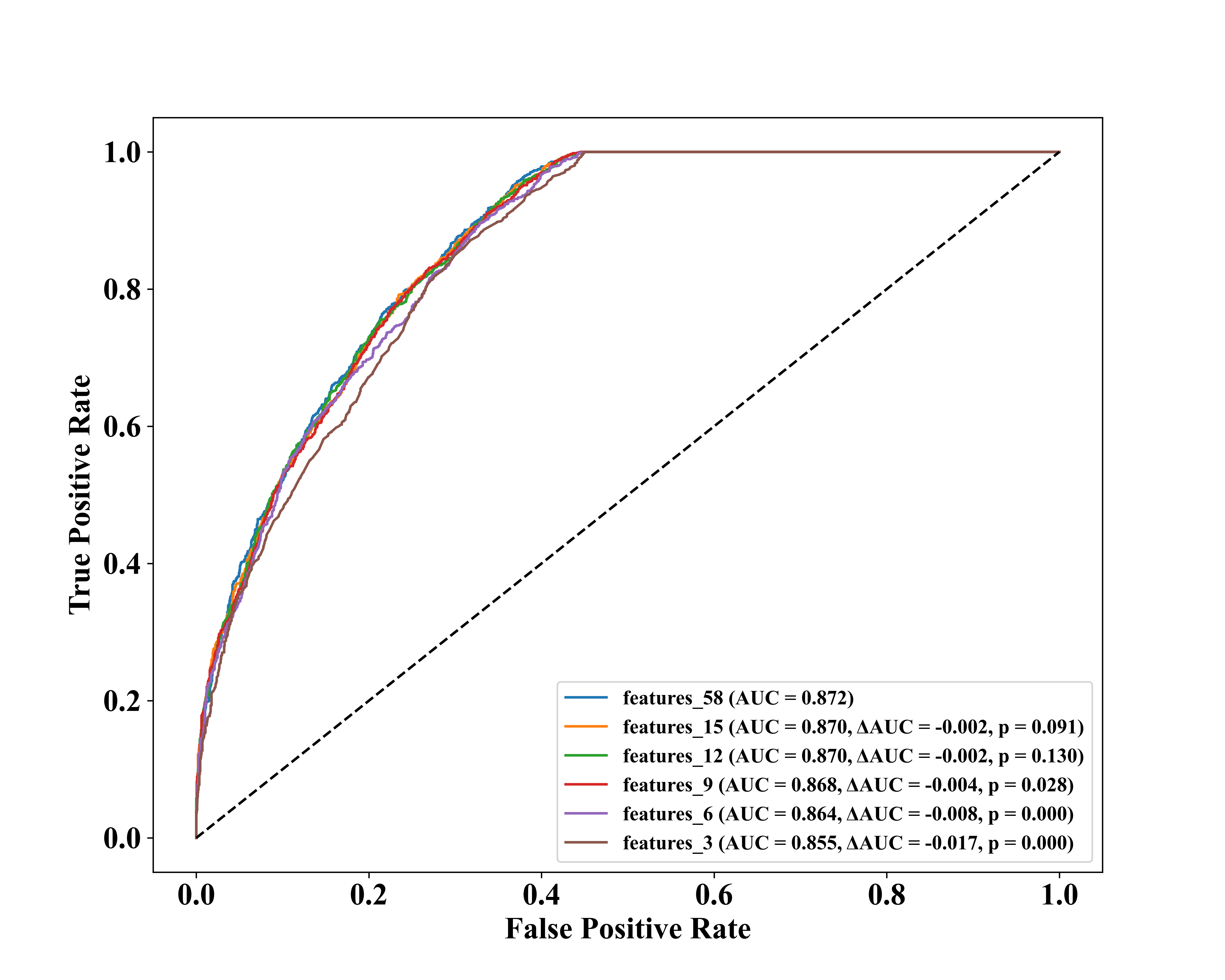

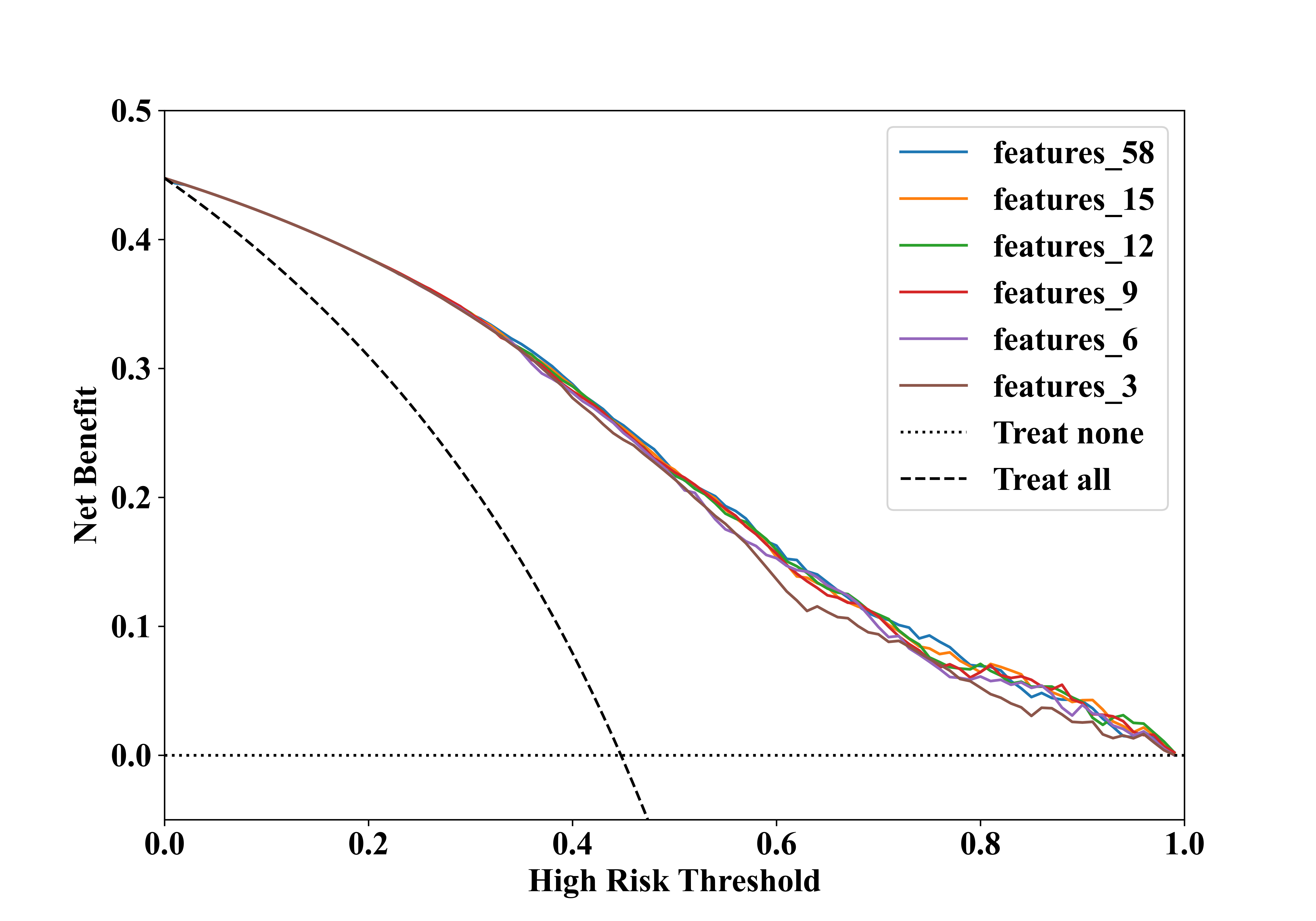


E

D

B

A

C


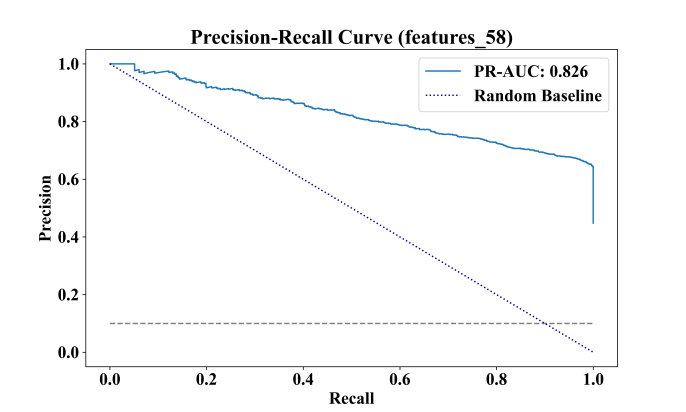

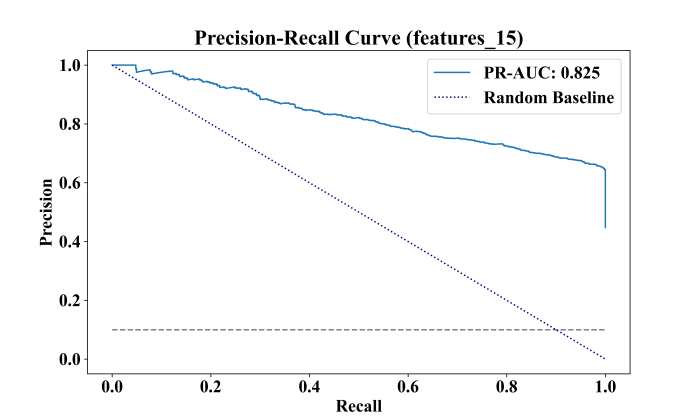

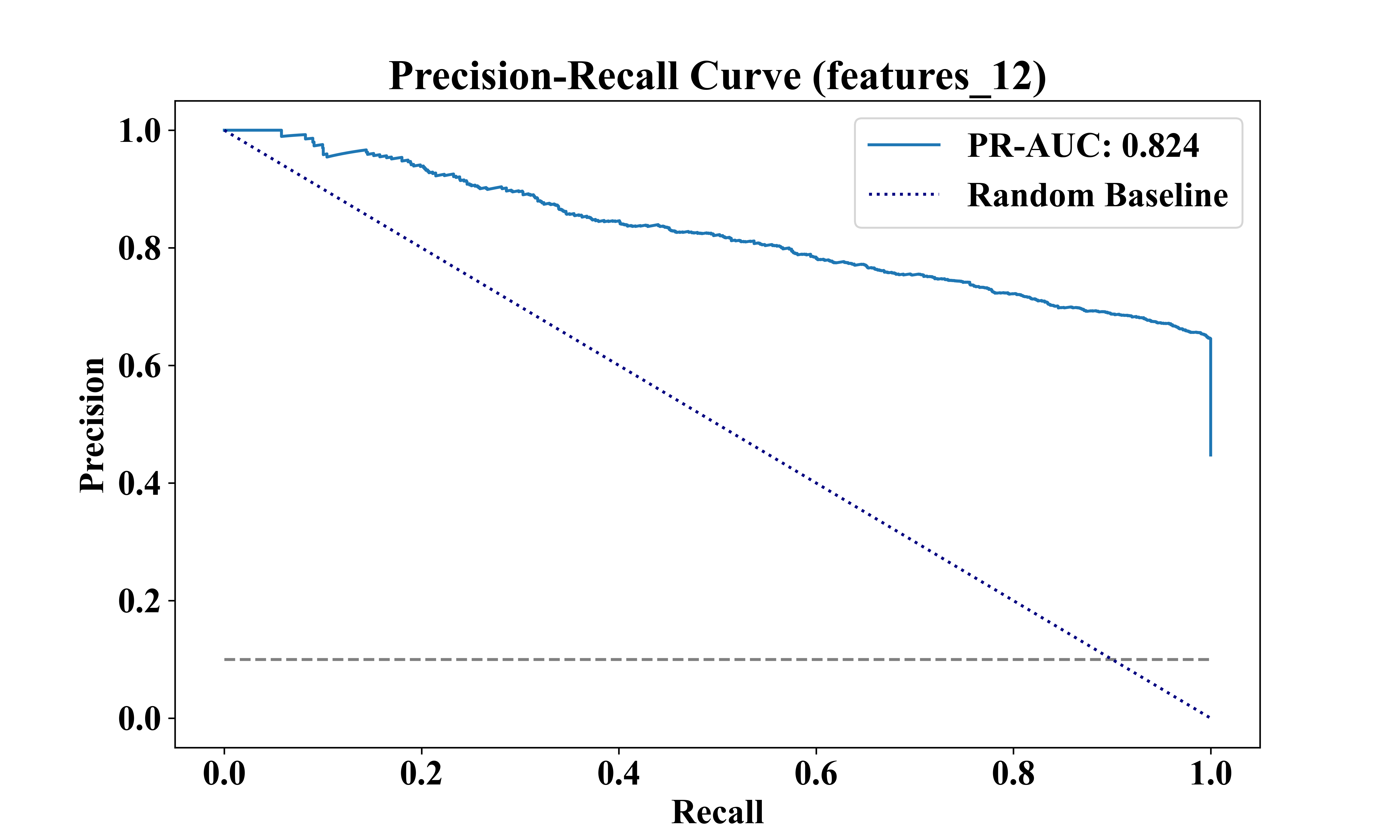

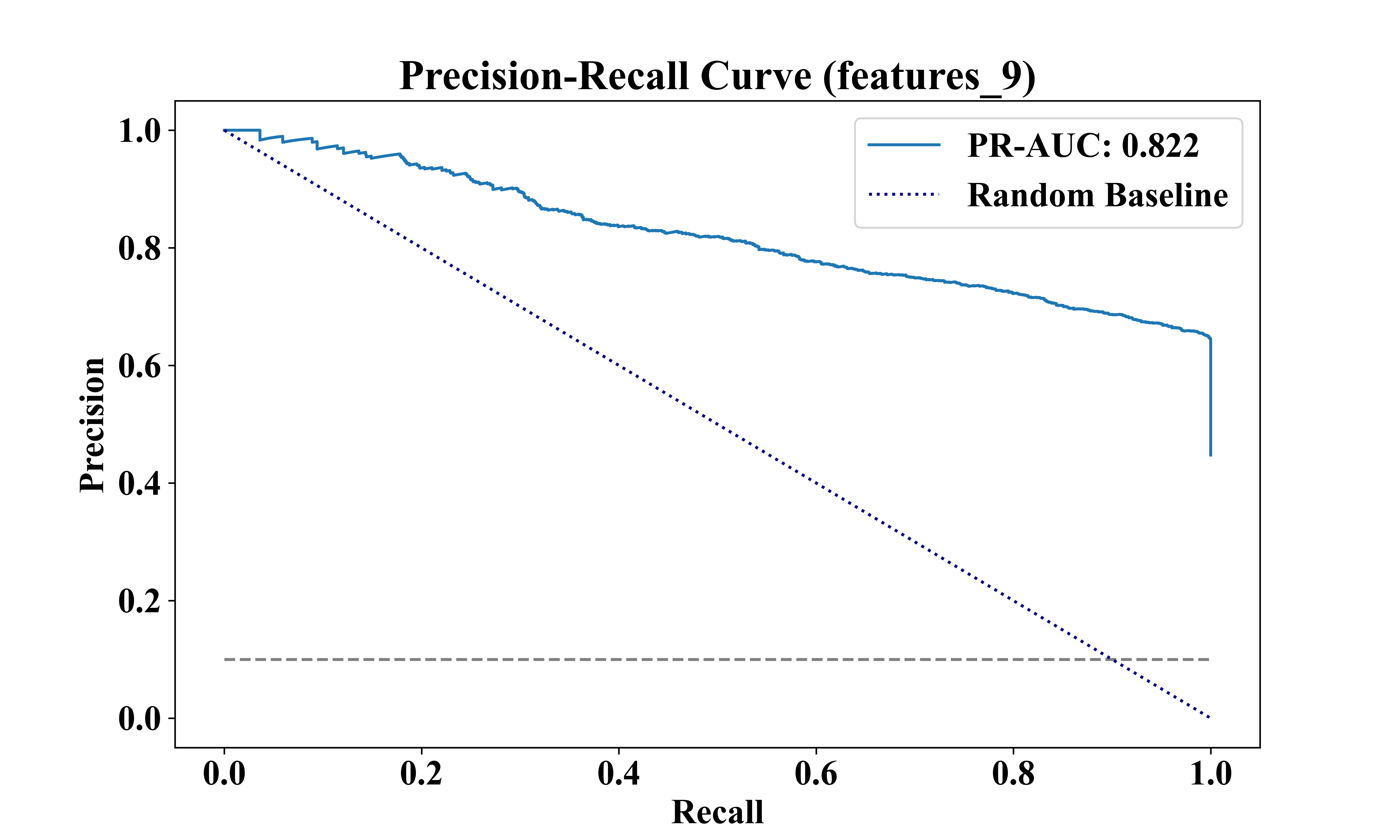

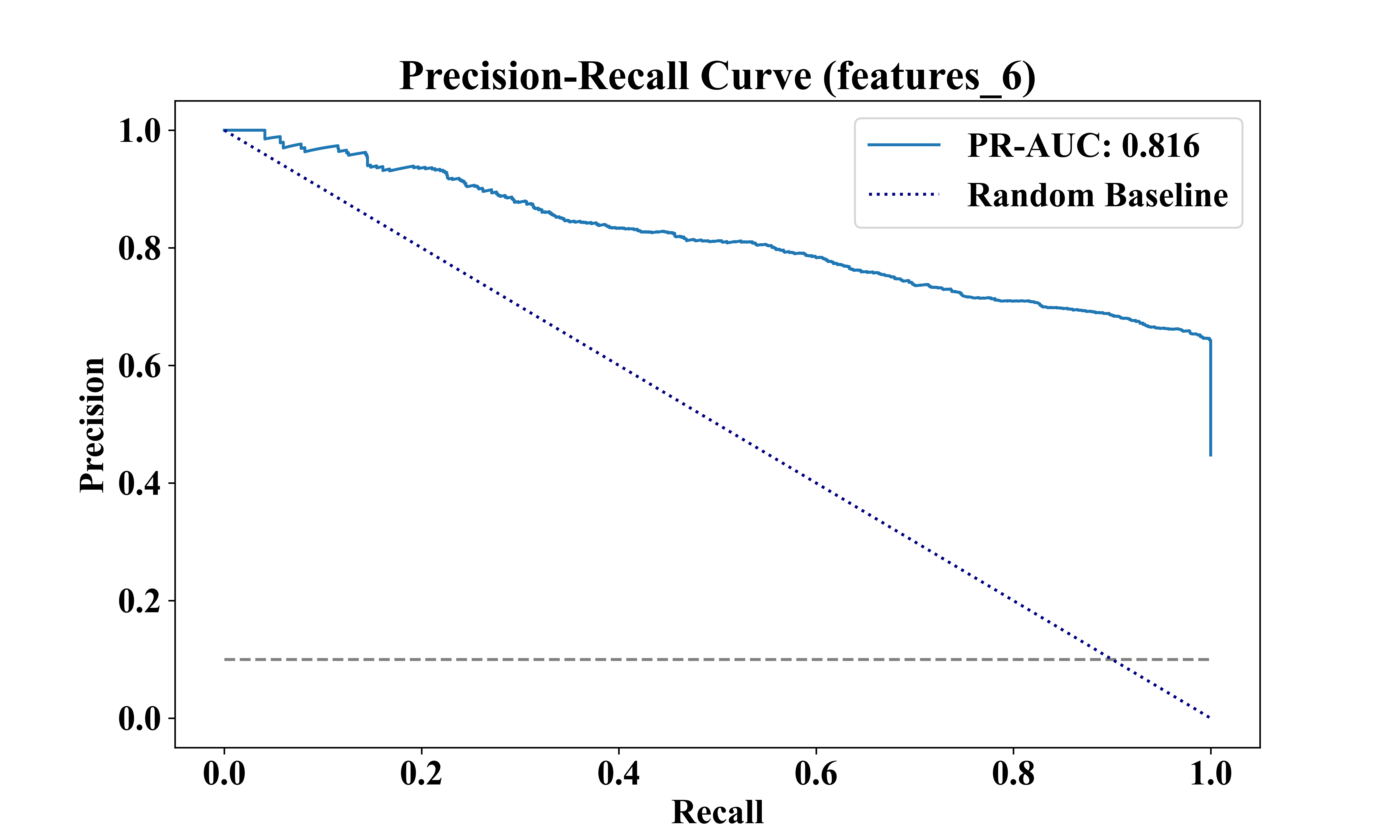

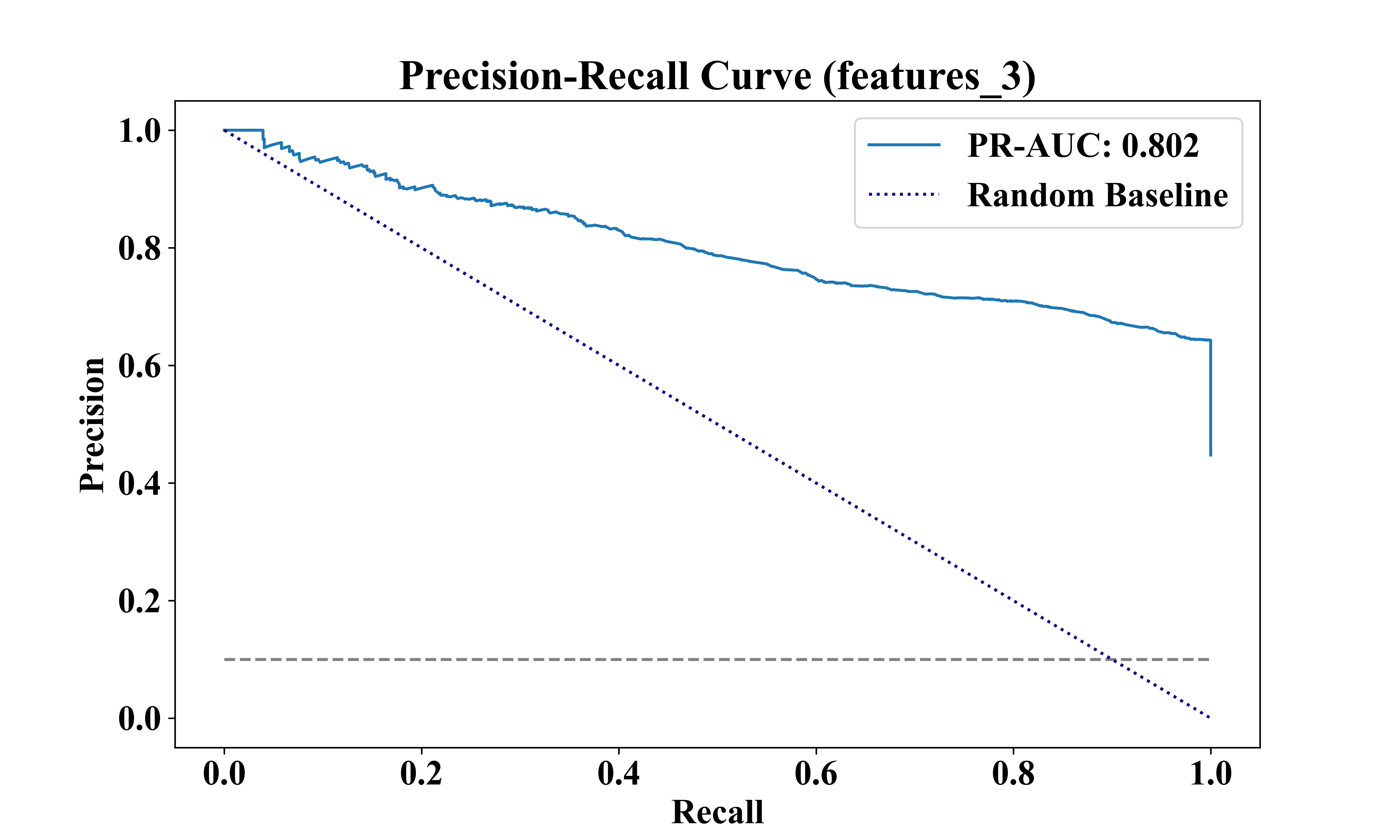


F

G

F

**Multimedia Appendix 7. Predictive performance of the gradient boosting machine model after reducing features.** (A-B) ROC curves (A) and DCA curves (B) of the GBM model with different features. ΔAUC and P value represented the comparison with the GBM model with 58 features. (C-G) P-R curves of the RF model with 58 features (C), 15 features (D), 12 features (E), 9 features (F), and 6 features (G),6 features (F). These plots represented the predictive performance in the internal validation cohort. AUC: area under the ROC curve; DCA: decision curve analysis; P-R: precision-recall; GBM: gradient boosting machine; ROC: receiver-operating-characteristic.
