# Supplementary material for: Explainable Machine Learning Model for Predicting Persistent Sepsis-Associated Acute Kidney Injury: Development and Validation Study
Source: J Med Internet Res. 2025 Apr 28;27:e62932. doi: 10.2196/62932 (PMC12070005; doi:10.2196/62932)
Supplement: Multimedia Appendix 8 [file jmir_v27i1e62932_app8.docx]

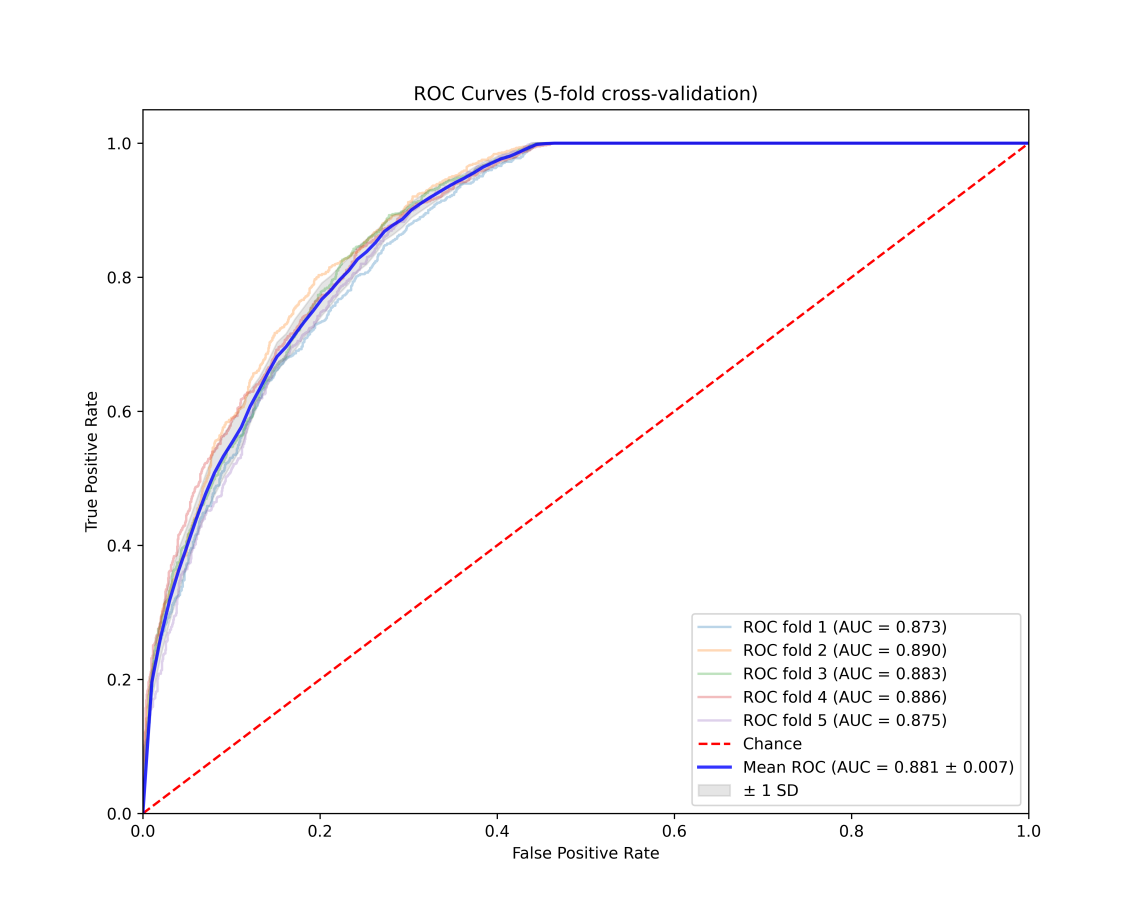

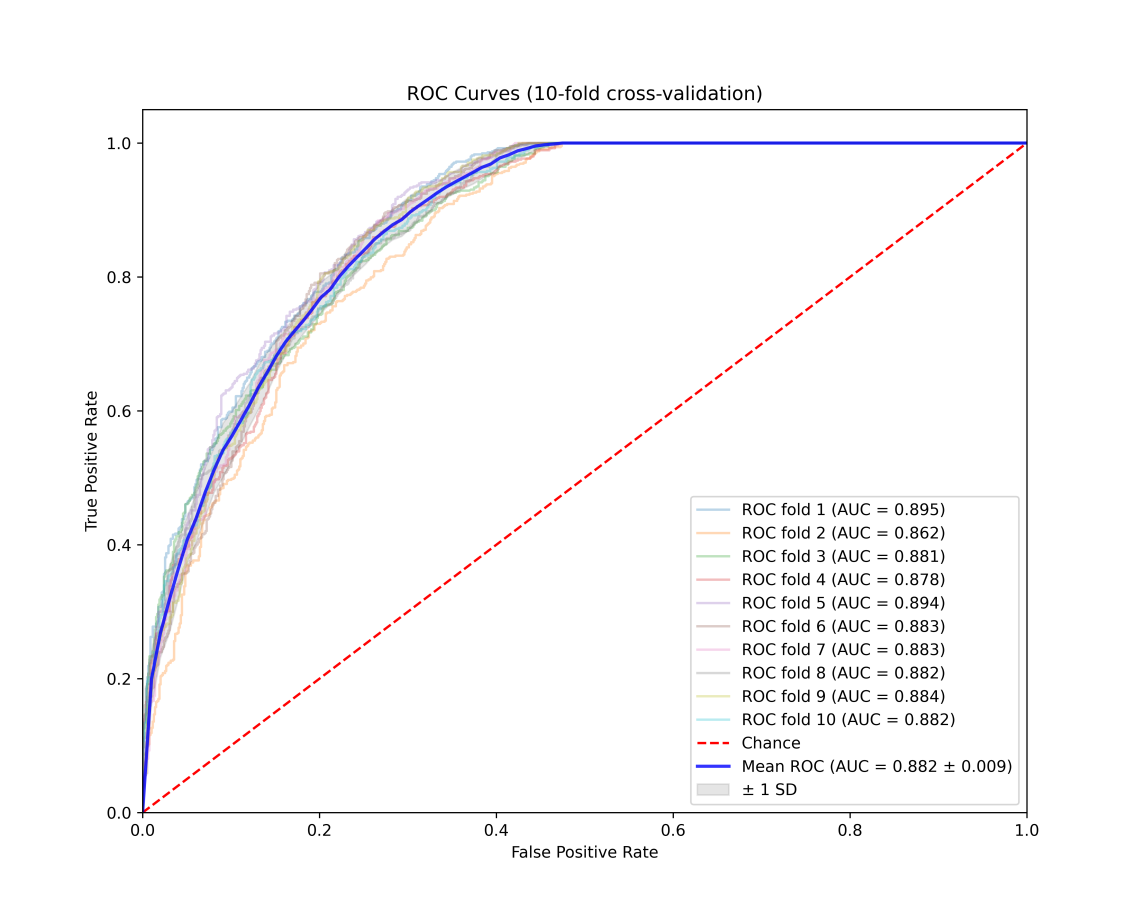


A

B

**Multimedia Appendix 8. Predictive performance of the final gradient boosting machine model in the cross-validation.**

(A-B) Five-fold (A) and ten-fold (B) cross validation of the final GBM model with 9 features. These plots represented the predictive performance of the cross validation in the derivation cohort. AUC: area under the ROC curve; GBM: gradient boosting machine; ROC: receiver-operating-characteristic; SD: standard deviation.
