# Supplementary material for: Explainable Machine Learning Model for Predicting Persistent Sepsis-Associated Acute Kidney Injury: Development and Validation Study
Source: J Med Internet Res. 2025 Apr 28;27:e62932. doi: 10.2196/62932 (PMC12070005; doi:10.2196/62932)
Supplement: Multimedia Appendix 9 [file jmir_v27i1e62932_app9.docx]

| 001  002  003  004  005  006  007  008  009  010  011  012  013  014  015  016  017  018  019  020  021  022  023  024  025  026  027  028  029  030  031  032  033  034  035  036  037  038  039  040  041  042  043  044  045  046  047  048  049  050  051  052  053  054  055  056  057  058  059  060  061  062  063  064  065  066  067  068  069  070  071  072  073  074  075  076  077  078  079  080  081  082  083  084  085  086  087  088  089  090  091  092  093  094  095  096  097  098  099  100  101  102  103  104  105  106  107  108 | **import** streamlit as st  **import** joblib  **import** pandas as pd  **import** shap  **import** matplotlib.pyplot as plt    # Load the trained model  model **=** joblib.load('model.joblib')    # Get the feature names for model training (make sure they are in the same order)  feature_names **=** model.feature_names_in_ **if** hasattr(model, 'feature_names_in_') **else** [      'aki_stage', 'creat_Δ', 'urineoutput', 'furosemide_dose_mg',      'BMI', 'sofa', 'rrt', 'mechvent', 'age',      'lactate_max', 'bun_max', 'pt_max'  ]    # Initializes the SHAP interpreter  explainer **=** shap.TreeExplainer(model)    # Set a class threshold  THRESHOLD **=** 0.384    **def** user_input_features():      col1, col2, col3 **=** st.columns(3)        with col1:          aki_stage **=** st.number_input("AKI Stage", value**=**3.0, format**=**"%.1f")          creat_delta **=** st.number_input("ΔCreatinine", value**=**2.0, format**=**"%.2f")          urineoutput **=** st.number_input("Urine Output (mL)", value**=**500.0, format**=**"%.1f")          furosemide_dose_mg **=** st.number_input("Furosemide Dose (mg)", value**=**0.0, format**=**"%.1f")        with col2:          bmi **=** st.number_input("BMI", value**=**27.0, format**=**"%.1f")          sofa **=** st.number_input("SOFA Score", value**=**5, format**=**"%d")          lactate_max **=** st.number_input("Lactate (mmol/L)", value**=**1.5, format**=**"%.1f")          bun_max **=** st.number_input("BUN (mg/dL)", value**=**13.0, format**=**"%.1f")        with col3:          age **=** st.number_input("Age", value**=**40, format**=**"%d")          rrt **=** st.selectbox("KRT", ["No", "Yes"])          mechvent **=** st.selectbox("Mechanical Ventilation", ["No", "Yes"])          pt_max **=** st.number_input("PT (sec)", value**=**12.0, format**=**"%.1f")        # Convert Yes/No to 1/0      rrt **=** 0 **if** rrt **==** "No" **else** 1      mechvent **=** 0 **if** mechvent **==** "No" **else** 1        # Create the DataFrame in the order of the features in which the model was trained      data **=** {          'aki_stage': aki_stage,          'creat_Δ': creat_delta,          'urineoutput': urineoutput,          'furosemide_dose_mg': furosemide_dose_mg,          'BMI': bmi,          'sofa': sofa,          'rrt': rrt,          'mechvent': mechvent,          'age': age,          'lactate_max': lactate_max,          'bun_max': bun_max,          'pt_max': pt_max      }        # Make sure the column order is the same as when training  **return** pd.DataFrame([data], columns**=**feature_names)    **def** main():      st.title("Persistent SA-AKI Prediction Application")      st.write("## Please enter the details for Persistent SA-AKI prediction")        input_df **=** user_input_features()    **if** st.button("Predict"):  **try**:              prediction **=** model.predict(input_df)              prediction_proba **=** model.predict_proba(input_df)[0][1]              prediction **=** 1 **if** prediction_proba >**=** THRESHOLD **else** 0              st.write(f"### Prediction Result: {'Persistent SA-AKI' if prediction == 1 else 'No Persistent SA-AKI'}")              st.write(f"### Prediction Probability: {prediction_proba:.4f}")                # SHAP interpretation              shap_values **=** explainer(input_df)                # Create a SHAP force plot              plt.figure()              shap.force_plot(                  base_value**=**explainer.expected_value,                  shap_values**=**shap_values.values[0],                  features**=**input_df.iloc[0],                  feature_names**=**feature_names,                  matplotlib**=**True              )              st.pyplot(plt.gcf())              plt.clf()                st.info("""              **Tip:** The threshold of 0.384 was determined as the optimal cutoff point based on the Youden index (J statistic),              which maximizes both sensitivity and specificity. Cases with predicted probabilities ≥ 0.384 are classified as              persistent SA-AKI, while those below this threshold are classified as non-persistent SA-AKI.              """)    **except** Exception as e:              st.error(f"An error occurred: {str(e)}")              st.write("Input DataFrame columns:", input_df.columns.tolist())              st.write("Expected feature names:", feature_names)    **if** __name__ **==** '__main__':      main() |
| --- | --- |
